# Risk factors and short-term projections for serotype-1 poliomyelitis incidence in Pakistan: A spatiotemporal analysis

**DOI:** 10.1371/journal.pmed.1002323

**Published:** 2017-06-12

**Authors:** Natalie A. Molodecky, Isobel M. Blake, Kathleen M. O’Reilly, Mufti Zubair Wadood, Rana M. Safdar, Amy Wesolowski, Caroline O. Buckee, Ananda S. Bandyopadhyay, Hiromasa Okayasu, Nicholas C. Grassly

**Affiliations:** 1 Department of Infectious Disease Epidemiology, St Mary’s Campus, Imperial College London, London, United Kingdom; 2 World Health Organization (WHO), Islamabad, Pakistan; 3 Ministry of National Health Services, Regulations and Coordination, Islamabad, Pakistan; 4 Center for Communicable Disease Dynamics, Harvard T. H. Chan School of Public Health, Boston, Massachusetts, United States of America; 5 Department of Ecology and Evolutionary Biology, Princeton University, Princeton, New Jersey, United States of America; 6 Department of Epidemiology, Harvard T. H. Chan School of Public Health, Boston, Massachusetts, United States of America; 7 Bill & Melinda Gates Foundation, Seattle, Washington, United States of America; 8 World Health Organization (WHO), Geneva, Switzerland; National Institutes of Health, UNITED STATES

## Abstract

**Background:**

Pakistan currently provides a substantial challenge to global polio eradication, having contributed to 73% of reported poliomyelitis in 2015 and 54% in 2016. A better understanding of the risk factors and movement patterns that contribute to poliovirus transmission across Pakistan would support evidence-based planning for mass vaccination campaigns.

**Methods and findings:**

We fit mixed-effects logistic regression models to routine surveillance data recording the presence of poliomyelitis associated with wild-type 1 poliovirus in districts of Pakistan over 6-month intervals between 2010 to 2016. To accurately capture the force of infection (FOI) between districts, we compared 6 models of population movement (adjacency, gravity, radiation, radiation based on population density, radiation based on travel times, and mobile-phone based). We used the best-fitting model (based on the Akaike Information Criterion [AIC]) to produce 6-month forecasts of poliomyelitis incidence. The odds of observing poliomyelitis decreased with improved routine or supplementary (campaign) immunisation coverage (multivariable odds ratio [OR] = 0.75, 95% confidence interval [CI] 0.67–0.84; and OR = 0.75, 95% CI 0.66–0.85, respectively, for each 10% increase in coverage) and increased with a higher rate of reporting non-polio acute flaccid paralysis (AFP) (OR = 1.13, 95% CI 1.02–1.26 for a 1-unit increase in non-polio AFP per 100,000 persons aged <15 years). Estimated movement of poliovirus-infected individuals was associated with the incidence of poliomyelitis, with the radiation model of movement providing the best fit to the data. Six-month forecasts of poliomyelitis incidence by district for 2013–2016 showed good predictive ability (area under the curve range: 0.76–0.98). However, although the best-fitting movement model (radiation) was a significant determinant of poliomyelitis incidence, it did not improve the predictive ability of the multivariable model. Overall, in Pakistan the risk of polio cases was predicted to reduce between July–December 2016 and January–June 2017. The accuracy of the model may be limited by the small number of AFP cases in some districts.

**Conclusions:**

Spatiotemporal variation in immunization performance and population movement patterns are important determinants of historical poliomyelitis incidence in Pakistan; however, movement dynamics were less influential in predicting future cases, at a time when the polio map is shrinking. Results from the regression models we present are being used to help plan vaccination campaigns and transit vaccination strategies in Pakistan.

## Abstract 2

To inform future eradication efforts, Natalie Molodecky and colleagues investigate the determinants of historical poliomyelitis incidence in Pakistan.

## Author summary

**Why was this study done?:**

- Despite the tremendous progress achieved by the Global Polio Eradication Initiative, the target for global polio eradication in 2016 has passed, and substantial challenges remain.
- Pakistan currently provides a major obstacle to achieving global polio eradication, having contributed to 73% and 54% of globally reported poliomyelitis in 2015 and 2016, respectively.
- A better understanding of the key risk factors and underlying population movement dynamics driving the continued incidence of poliomyelitis in Pakistan would help to more accurately assess and predict polio risk so that vaccination strategies are optimal.

**What did the researchers do and find?:**

- We developed a statistical model to identify the key spatiotemporal risk factors and population movement dynamics driving the continued incidence of poliomyelitis in Pakistan and evaluated the performance of the model in forecasting wild poliovirus type 1 (WPV1) cases.
- Spatiotemporal variation in immunisation performance and population movement patterns are important determinants of poliomyelitis cases in Pakistan.
- The model forecasts of poliomyelitis incidence showed good predictive ability and an overall reduction in risk of polio cases in the first half of 2017.

**What do these findings mean?:**

- By understanding the spatiotemporal heterogeneities that contribute to the continued persistence of poliomyelitis in Pakistan, the program can move forward towards eradication with a more proactive and informed strategic approach to vaccination.
- Results from this model are being used to plan future vaccination campaigns and transit vaccination strategies in Pakistan.

## Introduction

The Global Polio Eradication Initiative (GPEI) has reached a defining moment. Only 37 cases of poliomyelitis associated with wild-type poliovirus (“WPV cases”) were reported in 2016—the lowest annual count since inception of the GPEI in 1988 [1].

Currently, only 3 countries remain endemic for poliomyelitis associated with WPV serotype-1: Pakistan, Afghanistan, and Nigeria. Many additional milestones have been reached, including the last naturally occurring isolation of serotype-2 WPV in 1999, the last reported case of poliomyelitis associated with serotype-3 in 2012, and the absence of any reported wild poliovirus type 1 (WPV1) cases in Africa outside of Borno, Nigeria (which reported 4 WPV1 cases with a date of onset in July–August 2016 and remains a challenge because of issues with security, inaccessibility, and weak surveillance), since August 2014 (in Somalia). Despite the tremendous progress achieved by the GPEI, the target for global polio eradication in 2016 has passed, and substantial challenges remain.

Continued transmission of WPV1 in Pakistan is a major obstacle to achieving global polio eradication. In 2014, Pakistan reported 85% of the global WPV1 cases (306 out of 359). The number of reported WPV1 cases declined in 2015 to 54, marking significant progress for the Pakistan polio program, and only 20 WPV1 cases were reported in 2016. However, poliovirus persists in Pakistan, with continued transmission across district, provincial, and national borders (mainly into Afghanistan). Without making significant progress in Pakistan, the GPEI’s trajectory to interrupt transmission of WPV in 2017 will not be achieved.

Vaccination through routine immunization (RI) and supplementary immunization activities (SIAs or campaigns) with the oral polio vaccine (OPV) form the key intervention strategy to interrupt poliovirus transmission in Pakistan. Through RI, children in Pakistan are expected to receive 4 OPV doses (at birth and at 6, 10, and 14 weeks of age) and, since 2015, one dose of IPV coadministered with the 14-week OPV dose [2]. A strong RI system makes poliovirus transmission difficult to sustain, as infants are reached at an early age, providing little opportunity for them to contribute to transmission. To supplement RI in areas with limited health infrastructure and poor coverage, wide-scale and frequent SIAs targeting children <5 years of age have been implemented. Achieving high coverage of RI and SIAs is critical to interrupting transmission.

Continued transmission of WPV1 in Pakistan is partly due to inaccessibility of children to vaccination, resulting from political instability and violence, particularly in the Federally Administered Tribal Area (FATA) and Khyber Pakhtunkhwa (KP) provinces and the city of Karachi [3,4]. In mid-2012, militant leaders announced a ban on polio vaccination in the districts of North and South Waziristan, where an estimated 350,000 children reside [3,5]. Following military intervention in 2014, a mass exodus of susceptible and infected children occurred out of North Waziristan, and there was a subsequent spread of poliovirus into neighbouring districts. Furthermore, violence targeting polio vaccinators often makes efforts to access children dangerous and can interrupt and compromise vaccination campaigns. In addition to the challenge of inaccessible populations and insecurity, a large proportion of children are missed during campaigns as a result of frequent population movement by nomadic groups, particularly Pashtun populations moving from FATA and KP and in and out of Karachi. These groups have the lowest vaccination coverage rates in Pakistan [2,6].

Compounding these issues in Pakistan is the poor immunogenicity of OPV in this population. The GPEI has relied on OPV to eliminate poliovirus transmission because of its low cost, ease of administration, and its ability to produce a strong mucosal immune response and indirectly immunize secondary contacts [6]. However, because of its lower immunogenicity and effectiveness in tropical developing countries [7–9], OPV must be administered multiple times to a high proportion of children to interrupt transmission. This is particularly true in Pakistan, where the estimated effectiveness of 1 dose of trivalent OPV (tOPV) against WPV1 poliomyelitis is 12.5% (95% confidence interval [CI] 5.6%–18.8%) [10]. Monovalent OPV (mOPV1 and mOPV3) and bivalent OPV (bOPV) were licensed in 2005 and 2009, respectively, and in Pakistan, the effectiveness of these vaccines against WPV1 poliomyelitis has been estimated to be higher than tOPV (23.4% [95% CI 10.4%–34.6%] per dose of bOPV and 34.5% [16.1%–48.9%] for mOPV1) [10]. Despite their greater efficacy, multiple doses of these vaccines are still required to ensure protection.

The GPEI and partner organizations have addressed these issues through monitoring of vaccination coverage, implementation of campaigns when safety allows, and the establishment of permanent vaccination posts at borders and transit posts to immunize migrant children [3]. These initiatives to address remaining issues in achieving sufficient vaccination coverage have been aided by substantial improvements in accessibility of children for vaccination as a result of ongoing military intervention and presence in FATA, particularly in North and South Waziristan. Moreover, there has been a focused implementation of SIAs in high-risk areas identified as having low immunisation coverage, undervaccinated or missed populations, and weak RI infrastructure. These districts are classified as high risk based on statistical modelling approaches considering spatially heterogeneous estimates of immunity and immunization performance and expert opinion by public health officials. All districts in Pakistan are assigned a risk score based on this methodology and are subsequently grouped into 4 tiers of risk. Based on this assessment of historic risk and resource availability, SIAs are planned and operationalized. However, there are still knowledge gaps about transmission dynamics of poliovirus in Pakistan that must be better understood in order to accurately assess and predict risk so that vaccination strategies are optimal. In particular, an understanding of the underlying population movement dynamics would better inform operational strategies such as targeted surveillance and vaccination, as well as implementation or strengthening of vaccination at key transit posts.

To date, no Pakistan subnational WPV1 risk prediction models have incorporated mobility patterns or explored an extensive range of potential predictors. Previous modelling work on poliovirus transmission has been used to inform risk of WPV1 regionally at the national [11] or—for Nigeria and India—at the subnational level [12–15]; however, although some of these analyses explored spatially heterogeneous mixing, none of these explored different movement models. Recent work on predicting poliovirus transmission through exploring mobility patterns in Nigeria demonstrated variable success of different movement models in predicting WPV1 risk [16]. Moreover, incorporation of population movement into models of other infectious diseases has improved the predictive ability of forecasting incidence [17–20].

In this work, we estimated routine and supplementary immunisation coverage by district and 6-month period, population immunity to WPV1 poliomyelitis, and movement of poliovirus-infected individuals based on 6 different models, including 1 based on published mobile phone data [18], for Pakistan for the period of 2010–2016. We used regression models with defined time lags to correlate these variables with the incidence of WPV1 poliomyelitis by district. We used these models to identify the key risk factors driving the continued incidence of poliomyelitis in Pakistan and evaluated the performance of the best-fitting model in forecasting WPV1 cases over different 6-month periods from 2013–2016.

## Methods

### Data

#### Geodata

National, provincial, and district boundaries for Pakistan were obtained from the World Health Organization (WHO). The district-level administrative boundaries in Pakistan have changed over time. In order to examine trends over time, we retain the boundaries of districts in 2010. Additionally, we consider Karachi as 2 administrative units (based on historic polio epidemiology): (1) Gadap and Gulshan Iqbal and (2) the rest of Karachi. The number of districts considered in the model is 140. A map of Pakistan with the names of the 8 provinces is provided in Figure A in S1 Text.

#### Acute flaccid paralysis data

Acute flaccid paralysis (AFP) is described as a sudden onset of flaccid paralysis in 1 or more limbs and is characteristic of many aetiologies, such as Guillain-Barré syndrome, trauma, and enterovirus infections (including poliovirus) [21]. Globally, countries carry out nationwide surveillance programs to monitor cases of AFP, with reporting occurring through a network of healthcare providers [22]. All countries are expected to have an annual non-polio AFP rate of 1 per 100,000 population aged less than 15 years to meet global polio surveillance sensitivity indicators, with this rate increasing to 2 per 100,000 for endemic regions. All AFP cases are investigated, and detailed information is collected, including the province and district of residence; the dates of onset, notification, and stool collection; the age and sex of the individual; and the reported number of OPV doses received (with RI and SIA doses recorded separately in Pakistan). Moreover, stool samples are collected from AFP cases, and poliomyelitis cases are confirmed through isolation and sequencing of poliovirus. In our work, we used data for AFP cases with a clinical onset between January 2010 and December 2016.

#### SIA data

The National Polio Emergency Operations Center in Pakistan maintains a calendar of implemented and planned SIAs in Pakistan. The calendar includes district-level information on the dates of SIA implementation and the vaccine type used. We obtained data for the SIAs implemented or planned from January 2010 to June 2017.

### Statistical analyses

#### Estimation of potential covariates

RI coverage for each child was defined as the proportion of children receiving at least 3 OPV doses. SIA vaccination coverage for each child was calculated by dividing the reported number of OPV doses received through SIAs by the number of SIAs the child was expected to have experienced based on his or her date of birth, the date of paralysis onset, and the SIA calendar. Note that these estimates of RI and SIA coverage result in an average across the cohort of children present in the 6-month period and are not coverage estimates of RI and SIAs implemented only in the given 6-month period. Population immunity against poliomyelitis due to serotype-1 poliovirus for children aged <36 months was estimated based on the number of doses reported, the history of SIAs, and recent estimates of vaccine efficacy, using the methods described in [9]. Crude estimates of RI coverage, SIA coverage, and population immunity per district and 6-month period were obtained by taking the mean over the individual estimates from all non-polio AFP cases <36 months old in a given district and 6-month time period (18,544 total cases). To account for data sparsity, the crude estimates of RI coverage, SIA coverage, and population immunity were then spatially and temporally smoothed using a random-effects spatiotemporal model implemented using the R-INLA R package [23] (further details are given in Section S1.1 in S1 Text).

Geographic variation in the non-polio AFP rate, population size, population density, proportion of the population living in poverty, total births, temperature, and precipitation was also estimated. Further details are provided in Section S3.1 in S1 Text.

#### Estimation of the force of infection

The rate at which individuals are infected with poliovirus depends on the force (or hazard) of infection (FOI). This is a function of the rate at which individuals mix, and the conditions that make it more or less likely for virus transmission to occur (termed the transmission coefficient), multiplied by the number of infectious individuals at a given point in time. As there are a large number of asymptomatic poliovirus infections, we use a simplified version of the FOI that is based upon the number of reported poliomyelitis cases (which will likely capture where transmission intensity is highest). We simplify further by only considering whether a district will become infected to a level at which poliomyelitis will be reported rather than whether a susceptible individual will become infected. We assume that the transmission coefficient for within-district transmission does not vary spatially, and the log odds of a district reporting a poliomyelitis case from within district transmission is given as α_1_I_*j*,*t*−1_, where α_1_ is the within-districts transmission coefficient and I_j_ is the number of cases reported in the district in the previous 6 months. The log odds of a district j reporting a poliomyelitis case or poliomyelitis cases resulting from transmission from infections in district i is given as α_2_∑_i,i≠j_I_i,i≠j_S_ij_, where α_2_ is the between-district transmission coefficient, I_i_ is the number of cases reported in district i in the previous 6 months, and S_ij_ is the spatial component of movement from districts i to j, which may be 1 of 6 spatial models detailed below. In summary, therefore, the overall FOI determining the log odds of a district j reporting a poliomyelitis case or poliomyelitis cases is given as λ_j_ = α_1_I_j,t−1_ + α_2_∑_i,i≠j_I_i,i≠j_S_ij_.

To accurately capture the FOI between districts, we compared 6 spatial models to describe the connectivity between districts: (1) an adjacency model; (2) a radiation model; (3) a radiation model adapted to incorporate population density; (4) a radiation model adapted to incorporate travel times; (5) a gravity model with optimized parameter values; and (6) a gravity model fit to published mobile phone data [18] (models 1 and 4 were inspired by peer review). The first model assumes that movement between pairs of districts is a function of direct adjacency. The remaining 5 models assume that the movement of individuals between pairs of districts is a function of population size (or density) and Euclidian distance (or travel time), but the weightings differ between models [24]. The radiation model is dependent on the population size of the districts and the population within a circle radius equal to the Euclidean distance between the 2 populations [24]. The optimized gravity model is dependent on 3 unknown parameter exponents, which were optimized by maximizing the log likelihood returned from the univariable mixed-effects logistic regression model. The gravity model based on mobile phone data was dependent on 3 unknown coefficients, estimated by fitting a linear regression model to the daily average number of trips between pairs of districts, extracted from mobile phone records between 1 June and 31 December 2013 (previously published) [18]. A separate FOI was estimated for transmission within districts, based on incidence within the same district in the preceding 6-month period. Further details are given in Section S2.1 in S1 Text.

#### Logistic regression model

We fit a series of univariable generalized linear mixed-effects logistic regression models to routine surveillance data reporting the presence or absence of 1 or more WPV1 cases in each district of Pakistan for 6-month intervals from January–June 2010 to July–December 2016. A separate model was fit for each fixed effect, including district-level spatial covariates: population size, population density, number of births, poverty, and mean annual temperature and precipitation (constant); and population immunity, RI coverage, SIA coverage, number of SIA campaigns, and non-polio AFP rate (time varying). For all spatiotemporal variables (e.g., population immunity), the previous 6-month time-period estimates were used in order to capture a lag between the measurement and effect of these independent variables on poliomyelitis incidence. The FOI within districts and each of the 6 FOIs between district terms were also compared in analogous univariable analyses. Random intercepts of province, district, and 6-month time interval were included in the univariable models to better explain variability between observations. Further details and model formulation are given in Section S4.1 and Equation 6 in S1 Text.

We then fit a series of multivariable mixed-effects logistic regression models to the same data and considering all potential predictor variables. Only the best-fitting movement model from the univariable analysis was incorporated into the multivariable analysis. The most parsimonious yet best-fitting model was selected based on the Akaike Information Criterion (AIC) using a stepwise addition approach [25]. Random intercepts of province, district, and 6-month time interval were included in the multivariable models to better explain variability between observations. The univariable and multivariable regression models were fitted through maximum likelihood estimation. Further details are given in Section S4.1 in S1 Text.

To test the predictive ability of the best-fitting multivariable model, we conducted 6-month-ahead out-of-sample predictions from July–December 2013 onwards. In this process, the best-fit model identified by the multivariable analysis was refitted to subsets of data to determine its forecasting predictive ability. For the prediction of July–December 2013, the best-fit model was based on data from January 2010 to June 2013; similarly, for the prediction of January–June 2014, the best-fit model was based on data from January 2010 to December 2013. This was repeated for each subsequent 6-month time period. The predicted probability of observing a case in a particular district in the subsequent 6 months was compared with observed cases in that district in the subsequent 6-month period and evaluated using the area under the curve (AUC) of the receiver operating characteristic curve. Based on the multivariable best-fit model and data until December 2016, 6-month-ahead predictions for January–June 2017 were conducted.

## Results

### Incidence of wild poliomyelitis serotype-1 cases

**Fig 1:**
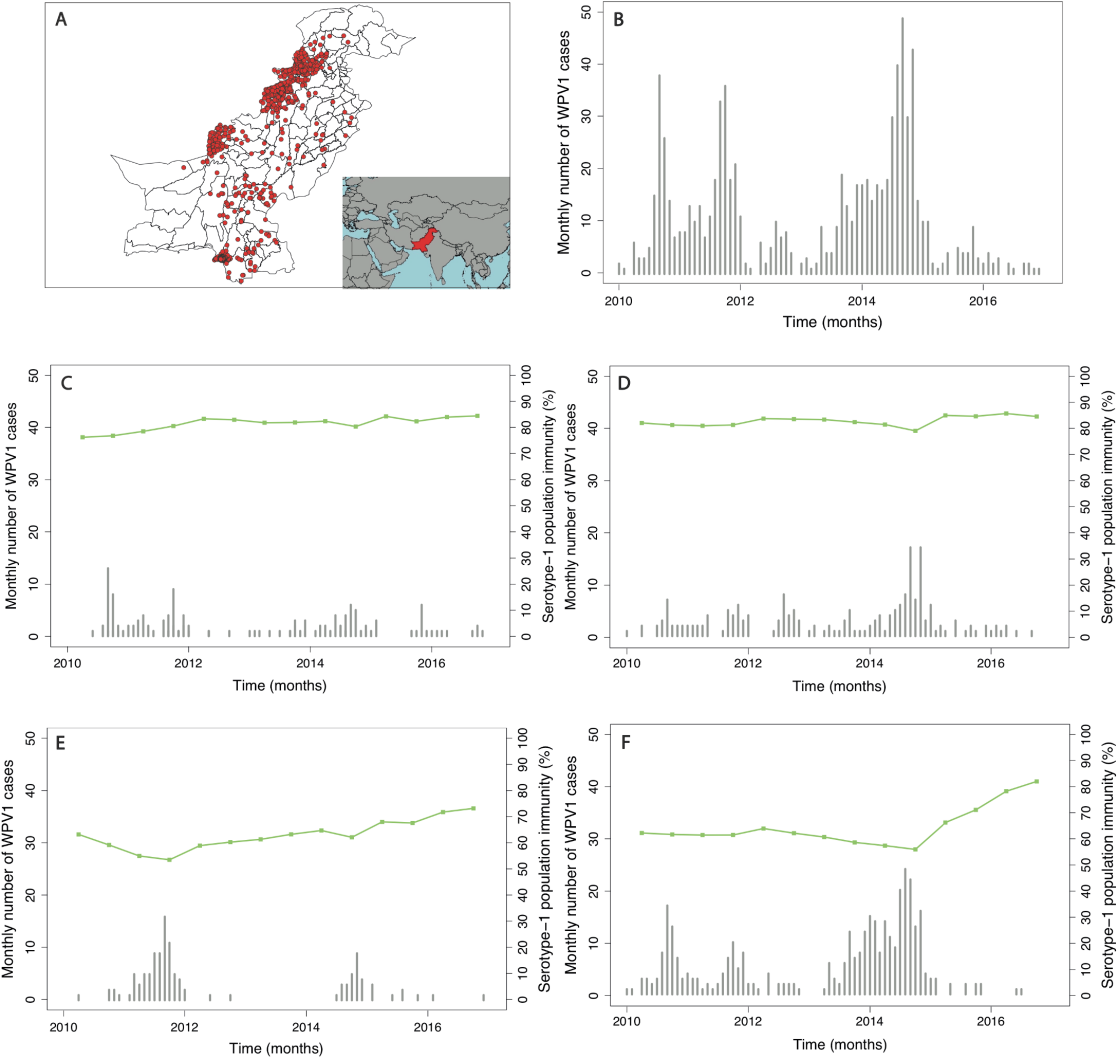
Spatial distribution and trends in the incidence of poliomyelitis over time in different regions of Pakistan. In (A), the spatial distribution of wild poliovirus type 1 (WPV1)-associated poliomyelitis cases in districts of Pakistan between January 2010 and December 2016 is shown (red dots). (B) Monthly confirmed WPV1-associated poliomyelitis cases in Pakistan reported between January 2010 and December 2016 are shown (bars). The same data are shown together with estimated serotype 1 vaccine-induced population immunity among children <36 months old (lines) for (C) Punjab, Sindh, Islamabad, Azad Jammu and Kashmir (AJK), and Gilgit-Baltistan, (D) Khyber Pakhtunkhwa, (E) Balochistan, and (F) the Federally Administered Tribal Area (FATA).

**Fig 2:**
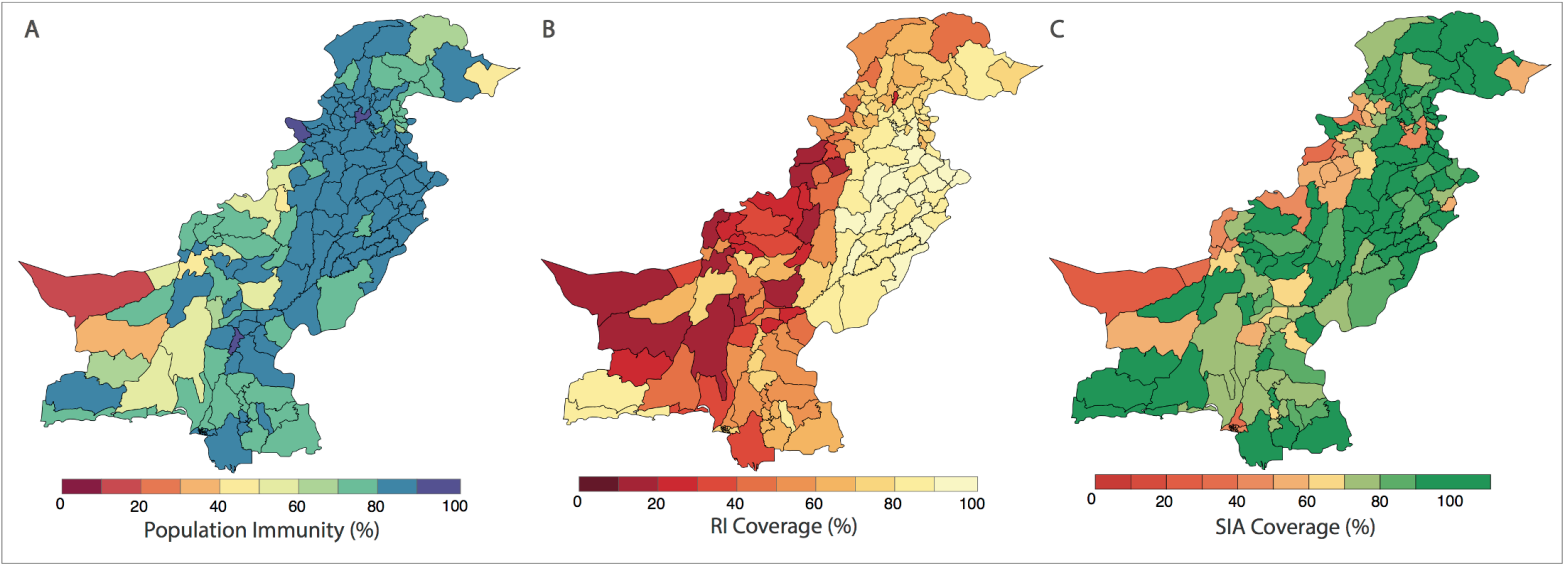
Spatial distribution of risk factors for wild poliovirus type 1 (WPV1)-associated poliomyelitis estimated from non-polio AFP data in districts of Pakistan for the period of July to December 2016. (A) Vaccine-induced population immunity against serotype-1 poliomyelitis for children <36 months old. (B) Routine immunization (RI) cohort coverage. (C) Supplementary immunization activity (SIA) cohort coverage (values >100% indicate more SIA doses were reported than expected given the SIA calendar). Complete figures with earlier time periods are included in S1 Text (Figures B, C, and E).

In Pakistan, the incidence of reported WPV1 cases steadily increased from 2010, reaching a peak in 2014. This surge in cases was followed by a sharp decline in 2015, which continued through 2016. The spatial distribution of cases has become more localised to higher-risk areas from 2012 onwards (Fig 1 and Figure K in S1 Text). Since 2010, cases have largely been concentrated in FATA, Balochistan, and Karachi (64% of the total cases between 2010 and 2016), where population immunity has been relatively low (median 64%, IQR 50%–74%) (Fig 2 and Figure E in S1 Text). There was a surge in cases between July to December 2010 and July to December 2011 (301 cases), with a subsequent decline until July to December 2013. In 2014, another surge in incidence occurred, with a reported 306 WPV1 cases, 218 of which came from FATA, Balochistan, and Karachi (295 including KP). Between July to December 2013 and July to December 2014, North Waziristan reported 70 cases, coinciding with a decline in population immunity. Between January 2010 and December 2016, Pakistan reported a total of 843 WPV1 cases.

### Univariable analysis

The probability of a district reporting at least 1 WPV1 case was associated with population immunity, RI coverage, SIA coverage, non-polio AFP rate, number of SIA campaigns, population size and density, annual number of births, and the FOI within and between districts (Table B in S1 Text). The spatial distribution of these covariates is presented in Fig 2 and Figures B to I in S1 Text.

The estimated FOI between districts based on the radiation model provided the best fit to the data compared with the 5 other models of population movement (adjacency, radiation based on population density, radiation based on travel times, gravity, and mobile-phone based; Table B in S1 Text).

### Multivariable analysis

The best-fitting model (AIC = 1,003.8) included fixed effects of RI coverage, SIA coverage, non-polio AFP rate, population size, and FOI (both within and between districts) (Table 1). Incorporating population immunity and the number of SIA campaigns in the previous 6-month time period as a categorical variable did not improve the fit, and therefore, they were removed from the final model. The model also included random intercepts of province and time point (i.e., 6-month intervals). We tested the random intercept of district nested within province; however, the variance tended towards 0 and was therefore excluded.

**Table 1:** Risk factors associated with the incidence of wild poliovirus type 1 (WPV1) cases based on the best-fitting multivariable mixed-effects lagged regression model for January–June 2010 through July–December 2016. The odds ratio (OR) and the 95% confidence interval (CI) for routine immunization and supplementary immunization activity (SIA) coverage are for an absolute 10% increase in these variables and a 1-unit increase for all other variables. Non-polio acute flaccid paralysis (AFP) rate is per 100,000 persons aged <15 years.

**Variable (fixed effects)**	**OR (95% CI)**	***P* value**
Routine immunization coverage (previous 6 months)	0.75 (0.67–0.84)	<0.001
SIA coverage (previous 6 months)	0.75 (0.66–0.85)	<0.001
Non-polio AFP rate (previous 6 months)	1.13 (1.02–1.26)	0.025
Log (population size)	2.62 (1.94–3.55)	<0.001
Cases in the same district (previous 6 months)	1.16 (1.04–1.28)	0.006
Cases in all other districts, weighted by probability of movement (previous 6 months; radiation model)	1.14 (1.02–1.27)	0.021
**Variable (random intercepts)**	**Variance**	**Standard Deviation**
Province	0.393	0.627
Year (6-month interval)	0.838	0.915

**Fig 3:**
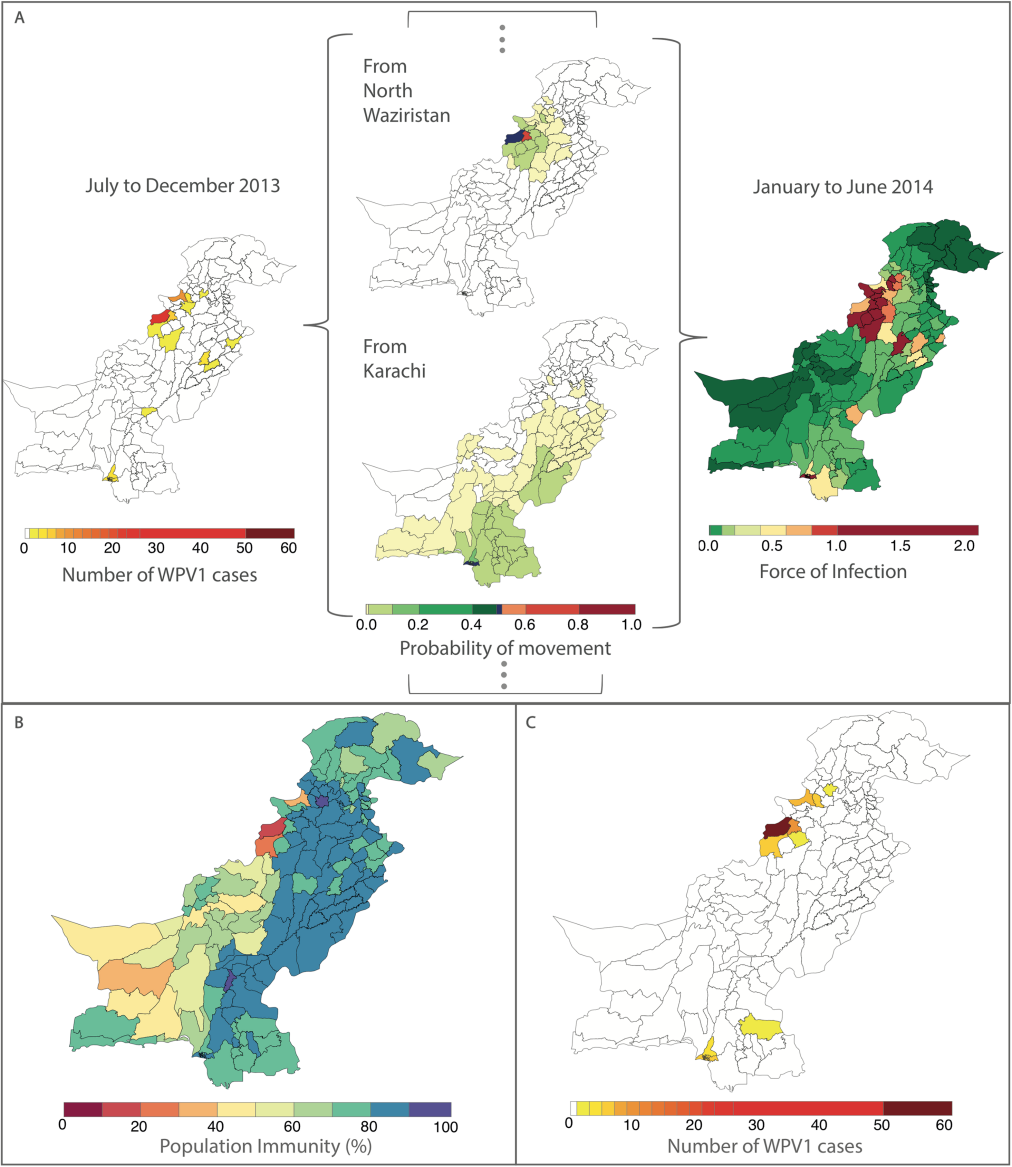
Illustration of the estimated force of infection (FOI) resulting from the movement of infected individuals between districts during January to June 2014. In (A), the components of the FOI are shown. Wild poliovirus type 1 (WPV1) cases in the previous 6 months (shown on the left) and estimated population movement calculated from the radiation model (shown for movement out of 2 chosen districts, centre, highlighted in dark blue) result in a district-specific FOI (right). The interplay between the FOI and the susceptibility of the population (population immunity, B) to determine the incidence of WPV1 cases in that 6-month period (C).

**Fig 4:**
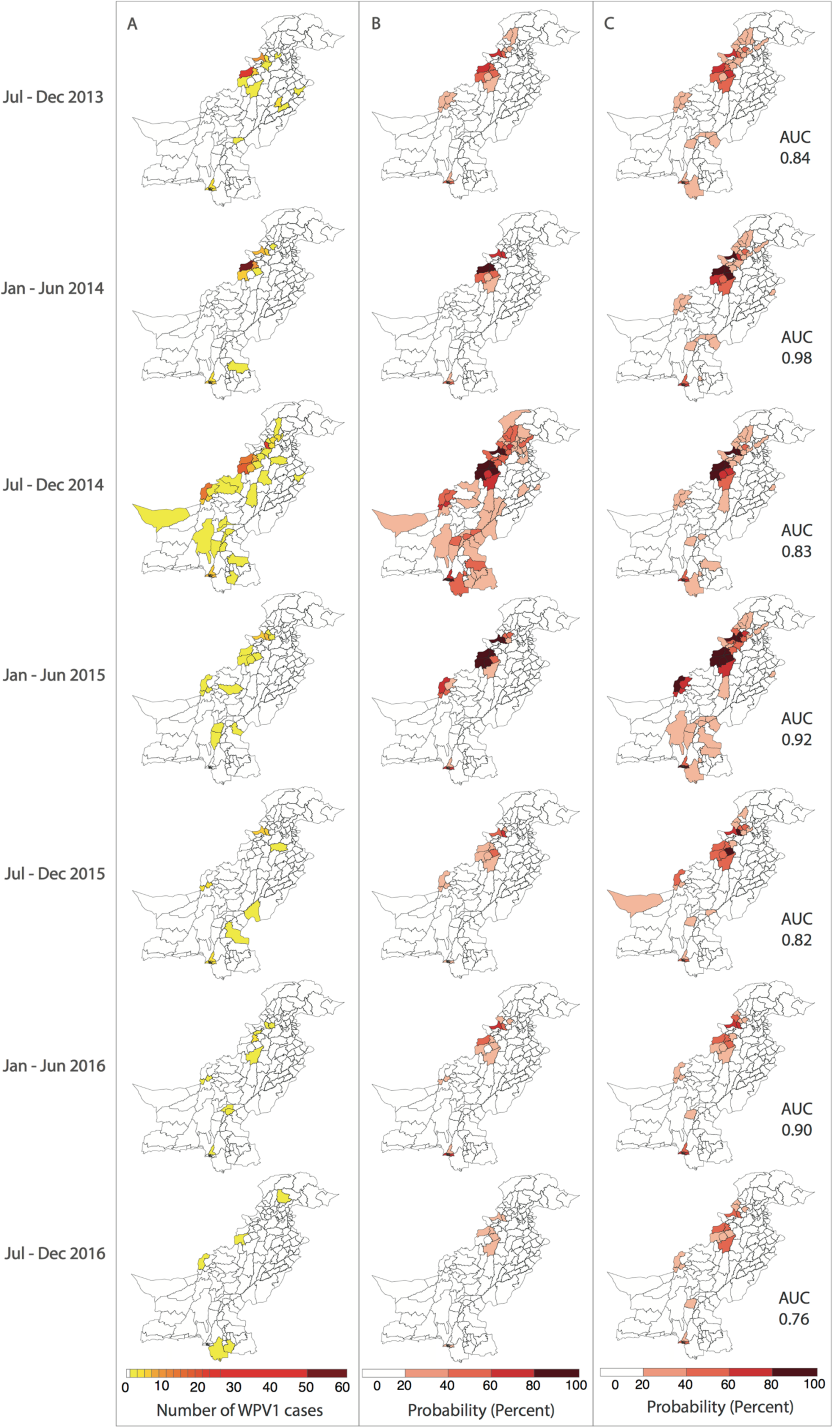
Reported and model-based estimates and forecasts of wild poliovirus type 1 (WPV1) cases between July 2013 and December 2016. (A) Observed WPV1 cases. (B) Estimated probability of reporting at least 1 WPV1 case based on the best-fit regression model including all available data (January 2010–December 2016). Complete figures with earlier time periods included in Figures K and L in S1 Text. (C) Predicted probability of reporting at least 1 WPV1 case for the same periods using data up to the end of the preceding 6-month period. AUC, area under the curve.

Removing the FOI from the multivariable model resulted in a significantly poorer fit (likelihood ratio test *P* value = 0.017). The radiation model (Figure I in S1 Text) captured both short- and long-distance movement, whereby short-distance movement was predicted to occur with a higher probability compared to long-distance movement (Figure J in S1 Text). The FOI was consistently high in and around FATA, Quetta Block, and Karachi, with periodic increases in Punjab and northern Sindh (Figure G in S1 Text). An illustration of the interplay between population immunity and between-districts FOI, with respect to WPV1 cases, is presented in Fig 3. The model-based estimates of the probability of a district reporting at least 1 WPV1 case closely correlate with the reported incidence of WPV1 cases (Fig 4A and 4B).

### Forecasts

The 6-month-ahead out-of-sample predictions of the probability of at least 1 reported WPV1 case for the period July to December 2013 through to July to December 2016 closely resemble the observed incidence of WPV1 cases for those periods (Fig 4A and 4C). The AUC ranged from 0.76 to 0.98 depending on the period examined, indicating that the model is able to reliably predict districts reporting cases (Figure M in S1 Text). The model consistently performed better in predicting the first half of the year (January–June) (AUC range: 0.90–0.98) when compared to the second half of the year (July–December) (AUC range: 0.76–0.84). Removing the FOI between districts (radiation model) from the forward projections resulted in a significantly poorer fit to the data from July–December 2014 onwards (Table C in S1 Text); however, there were no changes to the predictive ability of the model based on the AUC (Table D in S1 Text). Similarly, incorporating the FOI between districts based on the simpler adjacency model did not change the predictive ability (Table D in S1 Text).

**Fig 5:**
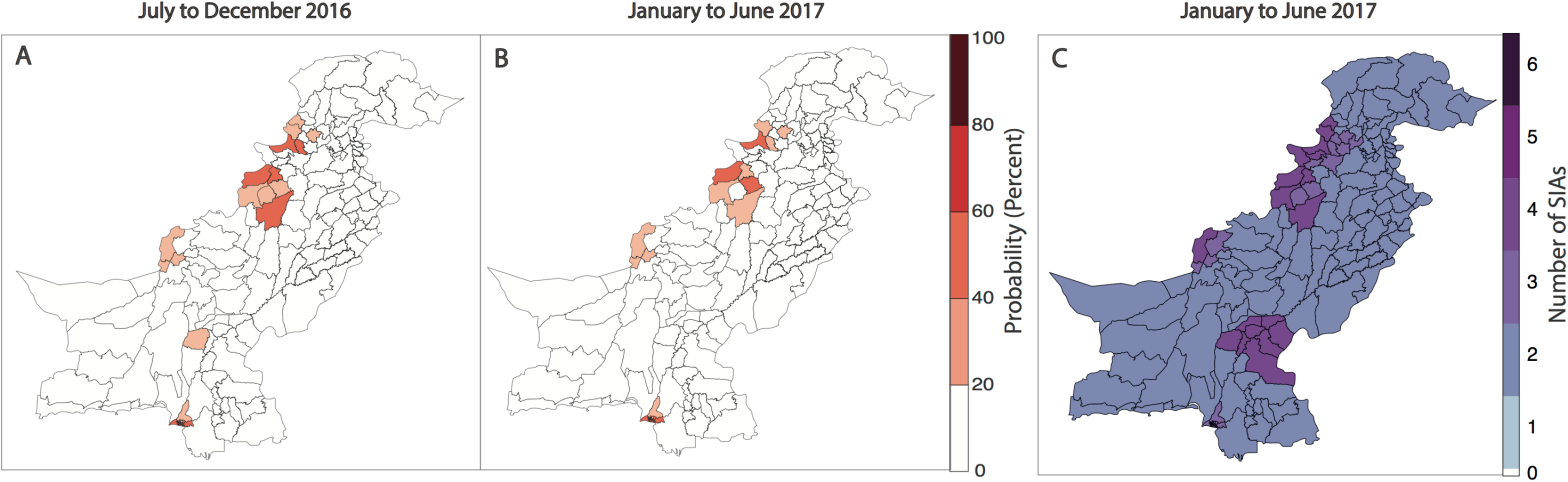
Forecasts of wild poliovirus type 1 (WPV1) poliomyelitis and vaccination response for the period July 2016 through June 2017. The estimated predicted probability of at least 1 WPV1 case for each district of Pakistan based on the best-fitting regression model is shown in (A) for July to December 2016 and (B) for January to June 2017. In (C), the planned supplementary immunization activity (SIA) calendar for January to June 2017 is shown based on national plans (as of January 2017).

Forecasts for January to June 2017 indicate that risk is concentrated in FATA, neighbouring KP, Quetta Block, and Karachi. In 9% of the districts, the probability of reporting a WPV1 case in the first half of 2017 was >20%, compared to 11% in the previous 6-month period and 13% in the first half of 2016 (Fig 5A, Fig 5B and Fig 4C). Between July to December 2016 and January to June 2017, the estimated probability of reporting at least 1 WPV1 case declined by >10% in 5 districts: in FATA (North Waziristan) and bordering areas of KP (Bannu, DI Khan, Peshawar, and Tank). There were no districts with increases >10%; however, 3 districts reported increases in risk between 2%–9% (Jafarabad, Lakki Marwat, and Killa Abdullah). Between the first and second half of 2016, the estimated probability of reporting at least 1 WPV1 case declined by >10% in 7 districts: in FATA (Bajour, Khyber, and Mohmand) and bordering areas of KP (Lakki Marwat and Peshawar) and both administrative units of Karachi (i.e., Karachi and Gadap/Gulshan Iqbal). There were no districts with increases >10%; however, 5 districts reported increases in risk between 2%–9% (Bannu, DI Khan, Shikarpur, Nasirabad, and Noshki). The predicted probability of reporting a serotype-1 case based on the model closely aligns with where SIA campaigns have been planned in January to June 2017 (as of January 2017) (Fig 5C).

## Discussion

Pakistan is currently one of only 3 remaining endemic countries reporting indigenous WPV1-associated poliomyelitis. Despite progress towards polio eradication, substantial challenges remain. In order to accurately assess risk and proactively implement effective vaccination strategies, a better understanding of the spatiotemporal heterogeneities and movement dynamics that contribute to transmission in Pakistan is essential.

We developed a statistical model that explores the relationship between reported WPV1 cases and potential covariates between 2010 and 2016 in Pakistan. The best-fit model was able to reliably forecast districts reporting at least 1 WPV1 case in the following 6-month period by identifying key spatial heterogeneities that contribute to poliovirus transmission in Pakistan. Moreover, the model identified an appropriate population movement structure that was able to capture the spatial patterns of reported WPV1 cases.

In our work, we found that the probability of reporting a WPV1 case significantly increased with decreasing RI and SIA coverage. In Pakistan, there has been little change in the spatial distribution of RI coverage over time, with a pronounced east–west dichotomy. RI coverage in Punjab has consistently remained above 80%, while estimates in FATA and Balochistan have remained very low at around 30%, providing an ideal environment for poliovirus persistence. Since 2015, there have been modest improvements in RI coverage nationally; however, estimates in most of the country (apart from Punjab) remain relatively low (S1.2 in S1 Text). These patterns are consistent with administrative and survey-based estimates.

Coverage of SIAs is likely to reflect issues with accessibility. In 2015, the implementation of new initiatives and a strengthened focus by the program to reach inaccessible children have resulted in substantial improvements in SIA coverage (S1.2 in S1 Text). This has been supported by the strengthened military presence in Pakistan, particularly in the areas bordering Afghanistan. Despite this progress, issues with security concerns continue to pose a challenge in key high-risk districts, particularly in FATA. Given the insufficient SIA coverage resulting from the prevalence of chronically undervaccinated groups in Pakistan [26] and its association with reported WPV1 cases, strengthening the quality of SIA campaigns is essential in mitigating risk of poliovirus transmission.

Population immunity was highly predictive of reporting WPV1 cases in the univariable analysis; however, as population immunity indirectly reflects RI and SIA coverage, it was redundant in the multivariable model. Patterns of RI coverage likely also reflect socioeconomic factors, such as health system infrastructure and health-seeking behaviour, whereas SIA coverage reflects issues with access, political instability, and cultural beliefs. Given that RI and SIA coverage likely capture additional information about demographic and socioeconomic factors, they provide additional information to the model and therefore, in combination, better explain the incidence of WPV1 cases than estimated vaccine-induced population immunity.

Similarly, in the univariable analysis, the number of SIA campaigns in the previous 6 months was highly predictive of WPV1 case reporting. Implementation of >1 SIA in a 6-month period significantly decreased the probability of a district reporting a case; however, this effect was dampened when the number of SIAs in a 6-month period surpassed 4. This is likely due to the focused implementation of SIAs in high-risk areas. Despite frequent SIA campaigns, these areas remain at risk because of continued, albeit decreasing, issues with access and the presence of chronically undervaccinated groups. The number of SIA campaigns in the previous 6 months was no longer predictive of poliovirus infection after accounting for the other covariates, particularly SIA coverage. Therefore, without addressing SIA coverage and reaching chronically undervaccinated and missed children, the impact of frequent SIAs on stopping poliovirus transmission is minimal.

The non-polio AFP reporting rate was significantly positively associated with WPV1 case incidence. This may reflect its value as an indicator of surveillance quality but also its association with the incidence of enterovirus infections and therefore, by extension, areas with efficient faecal-oral transmission of these viruses, including poliovirus. An association between increased surveillance quality and WPV1 cases may also be explained, in part, by the greater focus placed on areas of concern. For example, following the surge of WPV1 cases in North Waziristan in 2013–2014, the surveillance rate increased nearly 10-fold.

The FOI within districts (local) was significantly associated with WPV1 cases, reflecting the contribution of local transmission, which may be sustained in areas with consistently low immunisation coverage. Analysis of genetic sequence data has identified key poliovirus reservoirs in Karachi, Quetta, and Peshawar [27], whereby persistent virus circulation has been propagated by chronically undervaccinated groups. In addition, North Waziristan has been nearly consistently infected since the second half of 2009. The historically low coverage in North Waziristan has made it difficult to interrupt local transmission, creating a poliovirus reservoir that has perpetuated local transmission. However, access and the resulting immunization coverage have substantially improved in North Waziristan since 2014, with no reported WPV1 cases since May 2015.

In addition to the importance of local transmission within districts, importation of WPV1 from other districts and longer-distance transmission appear to play a key role in the persistence of polio in Pakistan. This is suggested by the significant association between the FOI estimated from the radiation model of population movement and the probability of observing WPV1 cases. Pakistani populations are highly mobile, with both short- and long-distance movement patterns, particularly in and out of Karachi [18]. These patterns of movement appear to be best captured by the radiation model [24], which outperformed gravity models fit to the WPV1 case data or to aggregated mobile phone records of population movement [18]. Similar results were found in a recent study on spatial dynamics and poliovirus transmission in Nigeria [16]. Moreover, although the radiation model largely captures short-distance movements, it outperforms the adjacency model because for some districts (e.g., out of Karachi, Quetta, and some other high-risk districts known to have long-distance movement patterns), the model predicts wide-scale cross-country movement.

The best-fitting model was evaluated to have a high sensitivity and specificity in predicting 6 months ahead of time those districts that would report WPV1 cases. The lowest predictive ability was in July to December 2016 (i.e., AUC of 0.76) and is likely attributed to 50% of the cases being reported from Sujawal and Badin, Southern Sindh (the first case was from Sujawal in September 2016). Sujawal is a newly formed district and is considered part of the Thatta district in the model. The high serotype-1 immunity and SIA coverage in Thatta masked the poor performance and subsequent risk of Sujawal. The lower predictive ability in the second half of each year (July–December) could be attributed to the more widespread circulation of poliovirus in the high season. Moreover, removing the FOI between districts (radiation model) from the forward projections resulted in a significantly poorer fit to the data from July–December 2014 onwards; however, there were no changes to the predictive ability of the model based on AUC. Therefore, the movement dynamics captured by the radiation model were important to obtain the best fit of our regression model to spatial patterns of poliomyelitis incidence but were not important for short-term projections in the context of declines in incidence and the spatial extent of transmission—in other words, at a time when the polio map is shrinking rather than spreading outwards. At this time, immunization performance may be more relevant than movement patterns in predicting the number and geographic location of cases, given that we have shown population immunity to have increased, therefore localising the virus. However, in the context of predicting the spread of emergent infections in highly susceptible populations, such as the recently detected serotype-2 vaccine-derived poliovirus in Quetta [28], the virus has the potential to spread rapidly, given that the OPV2 vaccine was globally withdrawn in April 2016 [29] and the number of susceptible individuals born since withdrawal is substantial. The underlying movement patterns in this context may therefore be more relevant. We are therefore currently developing a poliovirus transmission model that incorporates the movement model that we identify in the work presented here to help guide the response to this vaccine-derived poliovirus and potential future (re-)emergences of vaccine-derived and wild-type polioviruses.

Through the estimation of key risk factors and fit of a regression model to WPV1 case incidence data using these (lagged) risk factors, we have been able to reliably predict the probability of districts in Pakistan reporting WPV1 cases. However, there are some limitations to our study. Firstly, the estimates for population immunity, SIA coverage, and RI coverage were based on the recorded vaccination history of children with non-polio AFP, and we assume these estimates are representative of the entire population <36 months old. This assumption seems reasonable given the good correlation between WPV1 cases and estimates of population immunity, RI coverage, and SIA coverage. Recall error can affect the recorded vaccination history, with increased uncertainty of recall at higher doses (as demonstrated by SIA coverage estimates >100% in select district time periods). This is based on the assumption that accurate recall at high numbers is more difficult. Furthermore, the uncertainty in the estimates of population immunity, SIA coverage, and RI coverage was not accounted for in the model and may influence the reliability of the model. Additionally, the efficacy estimates used for OPV were based on analyses from case-control studies [10,30] to capture field settings and children in the cohorts at risk of poliomyelitis. Clinical trials have reported higher estimates of efficacy [31–33]; however, these studies have generally been performed in healthier populations in strict study settings. Where clinical studies have been conducted in high-risk communities [34–36], estimates are closer to those obtained from case-control studies [10,30,37]. However, population immunity (the only covariate based on vaccine efficacy) was not included in the final multivariable model, and therefore, the assumed vaccine efficacy did not have an impact on the forecasts. Moreover, although there was no clear trend in the temporal random intercept, there is some evidence that it may not be independent and identically distributed; further development of the temporal structure may be warranted. Secondly, our model predictions may have been improved by analysis of additional risk factors such as ethnicity (to capture long-distance connectivity in Pakistan [18,27]) or climatic variables; however, accurate and fine spatial resolution data on ethnicity and climate were not available. Thirdly, the mobile phone data used to estimate the parameters of the gravity model of population movement were unavailable in 29 districts (mainly in FATA and parts of Balochistan, where historic incidence of polio is greatest), due to lack of mobile phone tower coverage [18]. Therefore, the parameter estimates may not fully reflect the reliability of mobile phone data. Moreover, population movement based on the radiation model does not take into account temporal dynamics of mobility (e.g., resulting from sporadic events or seasonal patterns) or cross-border movement into Afghanistan. In future work, we aim to include an analysis of environmental surveillance data on poliovirus isolation and also potentially incorporate genetic information to allow better estimation of the patterns of poliovirus movement across Pakistan. We also plan to incorporate Afghanistan into the model to capture cross-border movement patterns, which are known to contribute to poliovirus transmission. Finally, we used only the incidence of poliomyelitis to inform our model and did not incorporate secondary OPV exposure. Currently, we are developing transmission models that consider underlying poliovirus infection and incorporate secondary spread of OPV.

The global eradication of polio is dependent upon stopping WPV1 transmission in Pakistan. By understanding the heterogeneities that contribute to continued persistence in Pakistan, the program can move forward in the endgame with a more proactive and informed strategic approach. In summary, we have developed a model to predict WPV1 cases 6 months ahead by exploring movement patterns and spatial heterogeneities that contribute to poliovirus transmission. In contrast to other risk models [11–13], this work is unique in that it identifies a reliable population movement structure that is able to capture transmission dynamics of poliovirus. We have provided the Pakistan polio program with updated results from this analysis since mid-2016. For the predictions in July–December 2016, the results from this model were used to help inform the spatial distribution of polio risk and the targeting of SIA campaigns and transit vaccination strategies in Pakistan. Encouragingly, we predict a lower overall probability of observing WPV1 cases in January to June 2017 compared with the previous 6-month period. Many high-risk districts, particularly in FATA, bordering areas of KP and Karachi, have demonstrated absolute declines in the risk of reporting cases. These declines in risk are largely driven by improvements in immunization performance and overall declining incidence in Pakistan. However, certain areas, particularly in southern KP, northern Sindh, and Quetta Block, are demonstrating a persistent, if not modestly increased, level of risk. Our forecast of WPV1 cases closely aligns with the spatial distribution of planned vaccination campaigns during the same period. We will continue to feedback the results from this model to the polio program to help inform polio risk and strategic targeting of vaccination in Pakistan. This risk-based approach to vaccination planning will be critical to the eventual elimination of WPV1 from Pakistan.

## Supporting information

S1 Checklist Reporting of Studies Conducted Using Observational Routinely-Collected Data (RECORD) statement. (DOCX)

S1 Text Supplementary information. (PDF)

## Abbreviations

AFP: acute flaccid paralysis
AIC: Akaike Information Criterion
AJK: Azad Jammu and Kashmir
AUC: area under the curve
bOPV: bivalent oral polio vaccine
CI: confidence interval
FATA: Federally Administered Tribal Area
FOI: force of infection
GPEI: Global Polio Eradication Initiative
KP: Khyber Pakhtunkhwa
mOPV: monovalent oral polio vaccine
OPV: oral polio vaccine
OR: odds ratio
RECORD: Reporting of Studies Conducted Using Observational Routinely-Collected Data
RI: routine immunization
SIA: supplementary immunization activity
tOPV: trivalent oral polio vaccine
WHO: World Health Organization
WPV: wild-type poliovirus
WPV1: wild poliovirus type 1
