# Supplementary material for: Risk factors and short-term projections for serotype-1 poliomyelitis incidence in Pakistan: A spatiotemporal analysis
Source: PLoS Med. 2017 Jun 12;14(6):e1002323. doi: 10.1371/journal.pmed.1002323 (PMC5467805; doi:10.1371/journal.pmed.1002323)
Supplement: S1 Checklist — (DOCX) [file pmed.1002323.s001.docx]

RECORD Statement—checklist of items that should be included in reports of observational studies

|  | Item No. | Recommendation  STROBE | Location in manuscript | Relevant text from manuscript | Recommendation  RECORD | Location in manuscript | Relevant text from manuscript |
| --- | --- | --- | --- | --- | --- | --- | --- |
| **Title and abstract** | 1 | (*a*) Indicate the study’s design with a commonly used term in the title or the abstract  (*b*) Provide in the abstract an informative and balanced summary of what was done and what was found | Title  Abstract,  paragraph 2-3 | *(a) Risk-factors and short-term projections for serotype-1 poliomyelitis incidence in Pakistan: a spatio-temporal analysis*  *(b) We fit mixed-effects logistic regression models to routine surveillance data recording the presence of poliomyelitis associated with wild-type 1 poliovirus in districts of Pakistan over 6-month intervals between 2010 to 2016. To accurately capture the force of infection (FOI) between districts, we compared six models of population movement (adjacency, gravity, radiation, radiation based on population density, radiation based on travel times, and mobile-phone based). We used the best-fitting model (based on the Akaike Information Criterion (AIC)) to produce 6-month forecasts of poliomyelitis incidence.*  *The odds of observing poliomyelitis decreased with improved routine or supplementary (campaign) immunisation coverage (multivariable odds ratio, OR = 0.75, 95% CI: 0.67-0.84 and 0.75, 0.66-0.85 respectively for each 10% increase in coverage) and increased with a higher rate of reporting non-polio acute flaccid paralysis (AFP) (OR=1.13, 1.02-1.26 for a 1-unit increase in non-polio AFP per 100,000 persons aged <15 years). Estimated movement of poliovirus infected individuals was associated with the incidence of poliomyelitis, with the radiation model of movement providing the best fit to the data. Six month forecasts of poliomyelitis incidence by district for 2013-2016 showed good predictive ability (area under the curve range: 0.76-0.98). However, although the best fitting movement model (radiation) was a significant determent of poliomyelitis incidence, it did not improve the predictive ability of the multivariable model. Overall, in Pakistan the risk of polio cases was predicted to reduce between July-December 2016 and January-June 2017. The accuracy of the model may be limited by the small number of AFP cases in some districts.* | 1.1: The type of data used should be specified in the title or abstract. When possible, the name of the databases used should be included  1.2: If applicable, the geographic region and timeframe within which the study took place should be reported in the title or abstract  1.3: If linkage between databases was conducted for the study, this should be clearly stated in the title or abstract | Abstract,  paragraph 2 | 1.1-1.2: *We fit mixed-effects logistic regression models to routine surveillance data recording the presence of poliomyelitis associated with wild-type 1 poliovirus in districts of Pakistan over 6-month intervals between 2010 to 2016.*  1.3: N/A |
|  |  |  |  |  |  |  |  |
| Background/  rationale | 2 | Explain the scientific background and rationale for the investigation being reported | Author Summary,  paragraph 1 | *Despite the tremendous progress achieved by the Global Polio Eradication Initiative, the target for global polio eradication in 2016 has passed and substantial challenges remain. Pakistan currently provides a major obstacle to achieving global polio eradication, having contributed to 73% and 54% of globally reported poliomyelitis in 2015 and 2016, respectively. A better understanding of the key risk factors and underlying population movement dynamics driving the continued incidence of poliomyelitis in Pakistan would help to more accurately assess and predict polio risk so that vaccination strategies are optimal.* |  |  |  |
| Objectives | 3 | State specific objectives, including any prespecified hypotheses | Introduction,  final paragraph | *In this work, we estimate routine and supplementary immunisation coverage by district and 6-month period, population immunity to WPV1 poliomyelitis, and movement of poliovirus infected individuals based on six different models including one based on published mobile phone data (18), for Pakistan for the period 2010-16. We use regression models with defined time-lags to correlate these variables with the incidence of WPV1 poliomyelitis by district. We use these models to identify the key risk factors driving the continued incidence of poliomyelitis in Pakistan and evaluated the performance of the best-fitting model in forecasting WPV1 cases over different 6-month periods from 2013-2016.* |  |  |  |
| Study design | 4 | Present key elements of study design early in the paper | Abstract,  paragraph 2 | *We fit mixed-effects logistic regression models to routine surveillance data recording the presence of poliomyelitis associated with wild-type 1 poliovirus in districts of Pakistan over 6-month intervals between 2010 to 2016. To accurately capture the force of infection (FOI) between districts, we compared six models of population movement (adjacency, gravity, radiation, radiation based on population density, radiation based on travel times, and mobile-phone based). We used the best-fitting model (based on the Akaike Information Criterion (AIC)) to produce 6-month forecasts of poliomyelitis incidence.*  Methods are provided in sequence in the methods section and further details are given in the supplement to allow clarity and reproducibility. |  |  |  |
| Setting | 5 | Describe the setting, locations, and relevant dates, including periods of recruitment, exposure, follow-up, and data collection | Introduction,  final paragraph | *In this work, we estimate routine and supplementary immunisation coverage by district and 6-month period, population immunity to WPV1 poliomyelitis, and movement of poliovirus infected individuals based on six different models including one based on published mobile phone data (17), for Pakistan for the period 2010-16. We use regression models with defined time-lags to correlate these variables with the incidence of WPV1 poliomyelitis by district. We use these models to identify the key risk factors driving the continued incidence of poliomyelitis in Pakistan and evaluated the performance of the best-fitting model in forecasting WPV1 cases over different 6-month periods from 2013-2016.* |  |  |  |
| Participants | 6 | (*a*) *Cohort study*—Give the eligibility criteria, and the sources and methods of selection of participants. Describe methods of follow-up  *Case-control study*—Give the eligibility criteria, and the sources and methods of case ascertainment and control selection. Give the rationale for the choice of cases and controls  *Cross-sectional study*—Give the eligibility criteria, and the sources and methods of selection of participants  (*b*) *Cohort study*—For matched studies, give matching criteria and number of exposed and unexposed  *Case-control study*—For matched studies, give matching criteria and the number of controls per case |  | N/A This is an observational study based upon all reported non-polio AFP cases <36 months in Pakistan. | 6.1: The methods of study population selection (such as codes or algorithms used to identify subjects) should be listed in detail. If this is not possible, an explanation should be provided  6.2: Any validation studies of the codes or algorithms used to select the population should be referenced. If validation was conducted for this study and not published elsewhere, detailed methods and results should be provided  6.3: If the study involved linkage of databases, consider use of a flow diagram or other graphical display to demonstrate the data linkage process, including the number of individuals with linked data at each stage |  | 6.1-6.3: N/A This is an observational study based upon all reported non-polio AFP cases <36 months in Pakistan. These were extracted from the Polio Information System at the World Health Organization. |
|  |  |  |  |  |  |  |  |
| Variables | 7 | Clearly define all outcomes, exposures, predictors, potential confounders, and effect modifiers. Give diagnostic criteria, if applicable | Methods, paragraph 2 | Diagnostic criteria:  *Acute flaccid paralysis (AFP) is described as sudden onset of flaccid paralysis in one or more limbs and is characteristic of many aetiologies, such as Guillain-Barré syndrome, trauma and enterovirus infections (including poliovirus) (20). Globally, countries carry out nationwide surveillance programs to monitor cases of AFP, with reporting occurring through a network of healthcare providers (21). All countries are expected to have an annual non-polio AFP rate of one per 100,000 population aged less than 15 years to meet global polio surveillance sensitivity indicators, with this rate increasing to two per 100,000 for endemic regions. All AFP cases are investigated and detailed information is collected, including: province and district of residence; the dates of onset, notification and stool collection; the age and sex of the individual; and the reported number of OPV doses received (with RI and SIA doses recorded separately in Pakistan). Moreover, stool samples are collected from AFP cases and poliomyelitis cases are confirmed through isolation and sequencing of poliovirus. In our work, we used data for AFP cases with clinical onset between January 2010 and December 2016.* | 7.1: A complete list of codes and algorithms used to classify exposures, outcomes, confounders, and effect modifiers should be provided. If these cannot be reported, an explanation should be provided |  | 7.1: N/A All reported non-polio AFP cases <36 months in Pakistan were included in the analysis. These were extracted from the Polio Information System at the World Health Organization. |
| Data sources/ measurement | 8* | For each variable of interest, give sources of data and details of methods of assessment (measurement). Describe comparability of assessment methods if there is more than one group | Methods, paragraph 1  Methods, paragraph 3 | *National, provincial and district boundaries for Pakistan were obtained from the World Health Organization (WHO).*  *The National Polio Emergency Operations Center in Pakistan maintains a calendar of implemented and planned SIAs in Pakistan. The calendar includes district-level information on the dates of SIA implementation and the vaccine type used. We obtained data for the SIAs implemented or planned from January 2010 to June 2017.* |  |  |  |
| Bias | 9 | Describe any efforts to address potential sources of bias |  | N/A |  |  |  |
| Study size | 10 | Explain how the study size was arrived at |  | N/A This is an observational study based upon OPV-dose histories from all reported non-polio AFP cases <36 months in Pakistan (see section above) |  |  |  |
| Quantitative variables | 11 | Explain how quantitative variables were handled in the analyses. If applicable, describe which groupings were chosen and why |  | N/A |  |  |  |
| Statistical methods | 12 | (*a*) Describe all statistical methods, including those used to control for confounding  (*b*) Describe any methods used to examine subgroups and interactions  (*c*) Explain how missing data were addressed  (*d*) *Cohort study*—If applicable, explain how loss to follow-up was addressed  *Case-control study*—If applicable, explain how matching of cases and controls was addressed  *Cross-sectional study*—If applicable, describe analytical methods taking account of sampling strategy  (*e*) Describe any sensitivity analyses | Methods, paragraph 4 | (a-b) Statistical methods are described in the main text and in detail in S1 Text.  (c) *To account for data sparsity, the crude estimates of RI coverage, SIA coverage and population immunity were then spatially and temporally smoothed using a random-effects spatio-temporal model implemented using the R-INLA R package (22) (further details are given in Section S1.1 in S1 Text).*  (d-e) N/A |  |  |  |
| Data access and cleaning methods |  |  |  |  | 12.1: Authors should describe the extent to which the investigators had access to the database population used to create the study population  12.2: Authors should provide information on the data cleaning methods used in the study  12.3: State whether the study included person-level, institutional-level, or other data linkage across two or more databases. The methods of linkage and methods of linkage quality evaluation should be provided |  | 12.1-12.3: The clean non-polio AFP data was extracted from the Polio Information System, which is maintained by WHO-Geneva. |
| Participants | 13* | (a) Report numbers of individuals at each stage of study—eg numbers potentially eligible, examined for eligibility, confirmed eligible, included in the study, completing follow-up, and analysed  (b) Give reasons for non-participation at each stage  (c) Consider use of a flow diagram | Methods, paragraph 4 | (a) *Crude estimates of RI coverage, SIA coverage and population immunity per district and 6-month period were obtained by taking the mean over the individual estimates from all non-polio AFP cases <36 months old in a given district and 6-month time period (18,544 total cases).*  (b-c) N/A all non-polio AFP cases <36 months were included. |  |  |  |
| Descriptive data | 14* | (a) Give characteristics of study participants (eg demographic, clinical, social) and information on exposures and potential confounders  (b) Indicate number of participants with missing data for each variable of interest  (c) *Cohort study*—Summarise follow-up time (eg, average and total amount) |  | (a-c) N/A |  |  |  |
| Outcome data | 15* | *Cohort study*—Report numbers of outcome events or summary measures over time  *Case-control study—*Report numbers in each exposure category, or summary measures of exposure  *Cross-sectional study—*Report numbers of outcome events or summary measures |  | (a-c) N/A |  |  |  |
| Main results | 16 | (*a*) Give unadjusted estimates and, if applicable, confounder-adjusted estimates and their precision (eg, 95% confidence interval). Make clear which confounders were adjusted for and why they were included  (*b*) Report category boundaries when continuous variables were categorized  (*c*) If relevant, consider translating estimates of relative risk into absolute risk for a meaningful time period | Methods, paragraph 8-9 | (a) Unadjusted estimates from the univariable analyses are presented in Table II of S1 Text. Estimates adjusted for confounding variables are presented in the main text Table 1. Details of methods for selecting final model are presented in the main text.  *We fit a series of univariable generalized linear mixed-effects logistic regression models to routine surveillance data reporting the presence or absence of one or more WPV1 cases in each district of Pakistan for 6-month intervals from January-June 2010 to July-December 2016. A separate model was fit for each fixed effect, including district-level spatial covariates: population size, population density, number of births, poverty and mean annual temperature and precipitation (constant); and population immunity, RI coverage, SIA coverage, number of SIA campaigns, non-polio AFP rate (time-varying). For all spatio-temporal variables (e.g. population immunity), the previous 6-month time-period estimates were used in order to capture a lag between the measurement and effect of these independent variables on poliomyelitis incidence. The FOI within districts and each of the six FOI between district terms were also compared in analogous univariable analyses. Random intercepts of province, district and 6-month time interval were included in the univariable models to better explain variability between observations. Further details and model formulation are given in Section S4.1 and Equation 6 in S1 Text.*  *We then fit a series of multivariable mixed-effects logistic regression models to the same data and considering all potential predictor variables. Only the best fitting movement model from the univariable analysis was incorporated into the multivariable analysis. The most parsimonious yet best fitting model was selected based on the Akaike Information Criterion (AIC) using a stepwise addition approach (24). Random intercepts of province, district and 6-month time interval were included in the multivariable models to better explain variability between observations. The univariable and multivariable regression models were fitted through maximum likelihood estimation. Further details are given in Section S4.1 in S1 Text.*  (b-c) N/A |  |  |  |
| Other analyses | 17 | Report other analyses done—eg analyses of subgroups and interactions, and sensitivity analyses |  | N/A |  |  |  |
| Key results | 18 | Summarise key results with reference to study objectives | Abstract, paragraph 3 | *The odds of observing poliomyelitis decreased with improved routine or supplementary (campaign) immunisation coverage (multivariable odds ratio, OR = 0.75, 95% CI: 0.67-0.84 and 0.75, 0.66-0.85 respectively for each 10% increase in coverage) and increased with a higher rate of reporting non-polio acute flaccid paralysis (AFP) (OR=1.13, 1.02-1.26 for a 1-unit increase in non-polio AFP per 100,000 persons aged <15 years). Estimated movement of poliovirus infected individuals was associated with the incidence of poliomyelitis, with the radiation model of movement providing the best fit to the data. Six month forecasts of poliomyelitis incidence by district for 2013-2016 showed good predictive ability (area under the curve range: 0.76-0.98). However, although the best fitting movement model (radiation) was a significant determent of poliomyelitis incidence, it did not improve the predictive ability of the multivariable model. Overall, in Pakistan the risk of polio cases was predicted to reduce between July-December 2016 and January-June 2017. The accuracy of the model may be limited by the small number of AFP cases in some districts.* |  |  |  |
| Limitations | 19 | Discuss limitations of the study, taking into account sources of potential bias or imprecision. Discuss both direction and magnitude of any potential bias | Discussion, paragraph 11 | *Through the estimation of key risk factors and fit of a regression model to WPV1 case incidence data using these (lagged) risk-factors we have been able to reliably predict the probability of districts in Pakistan reporting WPV1 cases. However, there are some limitations to our study. Firstly, the estimates for population immunity, SIA coverage and RI coverage were based on the recorded vaccination history of children with non-polio AFP, and we assume these estimates are representative of the entire population <36 months old. This assumption seems reasonable given the good correlation between WPV1 cases and estimates of population immunity, RI and SIA coverage. Recall error can affect the recorded vaccination history, with increased uncertainty of recall at higher doses (as demonstrated by SIA coverage estimates >100% in select district time periods). This is based on the assumption that accurate recall at high numbers is more difficult. Furthermore, the uncertainty in the estimates of population immunity, SIA coverage and RI coverage were not accounted for in the model and may influence the reliability of the model. Additionally, the efficacy estimates used for OPV were based on analyses from case-control studies [10, 30] to capture field settings and children in the cohorts at risk of poliomyelitis. Clinical trials have reported higher estimates of efficacy [31-33]; however, these studies have generally been performed in healthier populations in strict study settings. Where clinical studies have been conducted in high-risk communities [34-36], estimates are closer to those obtained from case-control studies [10, 30, 37]. However, population immunity (the only covariate based on vaccine efficacy) was not included in the final multivariable model and therefore, the assumed vaccine efficacy did not have an impact on the forecasts. Moreover, although there was no clear trend in the temporal random intercept, there is some evidence that it may not be independent identically distributed; further development of the temporal structure may be warranted. Secondly, our model predictions may have been improved by analysis of additional risk-factors such as ethnicity (to capture long distance connectivity in Pakistan [18, 27]) or climatic variables; however, accurate and fine spatial resolution data on ethnicity and climate were not available. Thirdly, mobile phone data used to estimate parameters of a gravity model of population movement were unavailable in 29 districts (mainly in FATA and parts of Balochistan, where historic incidence of polio is greatest), due to lack of mobile phone tower coverage [18]. Therefore, the parameter estimates may not fully reflect the reliability of mobile phone data. Moreover, population movement based on the radiation model does not take into account temporal dynamics of mobility (e.g. resulting from sporadic events or seasonal patterns) or cross-border movement into Afghanistan. In future work, we aim to include analysis of environmental surveillance data on poliovirus isolation and also potentially incorporate genetic information to allow better estimation of the patterns of poliovirus movement across Pakistan. We also plan to incorporate Afghanistan into the model to capture cross-border movement patterns which are known to contribute to poliovirus transmission. Finally, we used only the incidence of poliomyelitis to inform our model and did not incorporate secondary OPV exposure. Currently, we are developing transmission models that consider underlying poliovirus infection and incorporate secondary spread of OPV.* | 19.1: Discuss the implications of using data that were not created or collected to answer the specific research question(s). Include the discussion of misclassification bias, unmeasured confounding, missing data, and changing eligibility over time, as they pertain to the study being reported |  | 19.1: N/A |
| Interpretation | 20 | Give a cautious overall interpretation of results considering objectives, limitations, multiplicity of analyses, results from similar studies, and other relevant evidence | Discussion, paragraph 11 | *See paragraph immediately above* |  |  |  |
| Generalisability | 21 | Discuss the generalisability (external validity) of the study results |  | The manuscript is focused on identifying determinants and predicting risk of WPV1 in Pakistan and it is not applicable to generalise out of this context. |  |  |  |
| Funding | 22 | Give the source of funding and the role of the funders for the present study and, if applicable, for the original study on which the present article is based |  | This work was supported by the Bill and Melinda Gates Foundation (#OPP1099374), Medical Research Council (London, United Kingdom) (grants MR/J014362/1 and MR/K010174/1), Models of Infectious Disease Agent Study program (cooperative agreement 1U54GM088558) and James S. McDonnell Foundation  ASB is a Senior Program Officer in the polio team at the Bill & Melinda Gates Foundation. He was a technical resource, planner and manager for this project, but not a decision maker regarding funding |  |  |  |
| Accessibility of protocol, raw data, and programming code |  |  |  |  | 22.1: Authors should provide information on how to access any supplemental information such as the study protocol, raw data, or programming code |  | 22.1: Contact corresponding author. |

*Give information separately for cases and controls in case-control studies and, if applicable, for exposed and unexposed groups in cohort and cross-sectional studies.

**Note:** An Explanation and Elaboration article discusses each checklist item and gives methodological background and published examples of transparent reporting. The RECORD checklist is best used in conjunction with this article (freely available on the Web sites of PLoS Medicine at http://www.plosmedicine.org/, Annals of Internal Medicine at http://www.annals.org/, and Epidemiology at http://www.epidem.com/). Information on the RECORD Initiative is available at www.record-statement.org.
