# Supplementary material for: Risk factors and short-term projections for serotype-1 poliomyelitis incidence in Pakistan: A spatiotemporal analysis
Source: PLoS Med. 2017 Jun 12;14(6):e1002323. doi: 10.1371/journal.pmed.1002323 (PMC5467805; doi:10.1371/journal.pmed.1002323)
Supplement: S1 Text — (PDF) [file pmed.1002323.s002.pdf]

Supplementary Information

Risk-factors and short-term projections for serotype-1 poliomyelitis incidence in Pakistan: a spatio-temporal analysis

Natalie A Molodecky, Isobel M Blake, Kathleen M O'Reilly,
Mufti Zubair Wadood, Rana M Safdar, Amy Wesolowski, Caroline O Buckee,
Ananda S Bandyopadhyay, Hiromasa Okayasu, Nicholas C Grassly

Contents

S1 Estimating population immunity to serotype-1 poliovirus, routine immunisation (RI) coverage and supplementary immunisation activity (SIA) coverage 2

S1.1 Supplementary Materials and Methods . . . 2

S1.2 Supplementary Results . . . 3

S2 Estimation of force of infection (λ) 4

S2.1 Supplementary Methods . . . 4

S2.2 Supplementary Results . . . 5

S3 Estimation of potential covariates 6

S3.1 Supplementary Methods . . . 6

S3.2 Supplementary Results . . . 6

S4 Logistic regression model 7

S4.1 Supplementary Methods . . . 7

S4.2 Supplementary Results . . . 7

S5 Supplementary Figures and Tables 8

S1 Estimating population immunity to serotype-1 poliovirus, routine immunisation (RI) coverage and supplementary immunisation activity (SIA) coverage

S1.1 Supplementary Materials and Methods

Crude estimates of vaccine-induced population immunity by district and 6-month period

We computed crude estimates of serotype-1 population immunity in districts of Pakistan for children <36 months old in 6-month time intervals from Jan-Jun 2010 to Jul-Dec 2016. The individual crude estimates were obtained using the number of OPV doses received (accounting for the different serotype-1 OPV vaccines - tOPV, bOPV and mOPV1), n, and vaccine specific per-dose efficacy against serotype-1 previously estimated at $e_t = 0.125$, $e_b = 0.234$, $e_{m1} = 0.345$[1], proceeding as in previously published methods [2, 3]. The method assumes an all-or-nothing protective response to vaccination where the per-dose probability of inducing an immune response is independent of the previous doses. The probability that a child is protected is thus given by:

$$1 - (1 - e_t)^{n_t}(1 - e_b)^{n_b}(1 - e_{m1})^{n_{m1}} \tag{1}$$

where $e_{1,t}$, $e_{1,b}$ and $e_{1,m1}$ are the estimates of efficacy against serotype-1 per-dose of tOPV, bOPV and mOPV1 and n_t, n_b and n_{m1} are the estimated number of doses of tOPV, bOPV and mOPV1 the individual has been exposed to, based on the number of doses reported through SIAs and routine immunisation.

Because the reported number of SIA doses do not specify the vaccine type, we had to derive the number of vaccine specific OPV doses that a non-polio AFP case had received through SIAs. This was obtained by multiplying the number of reported doses received through SIA by the proportion of SIAs with each OPV type that the child was exposed to based on the SIA calendar. All routine doses in Pakistan were historically with tOPV. In April 2016, OPV2 was withdrawn and all subsequent RI doses were with bOPV.

The district 6-month crude estimate of serotype-1 population immunity was taken as the mean of individual estimates of protection adjusted by age in years (assuming equal weights of one year age groups).

Spatio-temporal models of population immunity, RI coverage and SIA coverage

To account for sparsity in the number of non-polio AFP cases by district 6-month period, we spatially and temporally smoothed our crude estimates of serotype-1 population immunity, RI coverage and SIA coverage from Jan-Jun 2010 to Jul-Dec 2016. This was done by fitting random-effects spatio-temporal models to the crude estimates using the Integrated Nested Laplace Approximation (INLA) approach [4] implemented in the R-INLA package [5], as in previously published methods [6]. This is a computationally efficient approach for Bayesian inference with latent Gaussian models to accurately approximating the posterior distribution [4].

The methods used to spatially and temporally smooth crude estimates of serotype-1 population immunity and RI coverage are described in detail in previously published work [6].

In contrast to the spatio-temporal model of population immunity and RI coverage described in [6], the model for smoothing SIA coverage assumed a gamma distribution due to the right-skewed tail of the data. Children reporting more doses than expected had SIA coverage estimates greater than 100%.

Let s_{ijt} be the crude estimate of SIA coverage in a district i (in a province j) at a 6-month period t, and n_{ijt} the number of non-polio AFP cases used to estimate s_{ijt}. We defined the following random-effects model for s_{ijt}:

$$s_{ijt} \sim \text{Gamma}\left(\eta_{ijt}, \frac{\sigma^2}{n_{ijt}}\right)$$

$$\text{logit}(\eta_{ijt}) = \alpha + \gamma_{it} + \delta_{jt} + \omega_t + \mu_i + \nu_i \quad (2)$$

where α is the intercept, γ_{it} and δ_{jt} are two space-time interactions at two different spatial levels (the district, γ, and the province, δ), ω_t is a temporal random effect that accounts for a general time trend, and μ_i and ν_i are respectively the spatial-unstructured and the spatial-structured random effects of the Besag, York and Mollié (BYM) model [7].

S1.2 Supplementary Results

For the spatio-temporal models of serotype-1 population immunity, RI coverage and SIA coverage, the intercept, α, the precision for the Gaussian observations* and for the two space-time interactions, as well as for the general time trend and for the BYM spatial components of the model were estimated (Table A). The smoothed estimates of serotype-1 population immunity, RI coverage and SIA coverage are presented in the main text (Fig 1 and Fig 2) and in Figure E, Figure B and Figure C. *Note that for SIA coverage, the gamma distribution was assumed.

Spatio-temporal distribution of SIA campaigns

The number of SIA campaigns with serotype-1 (i.e. tOPV, mOPV1 and bOPV) in Pakistan has remained consistent over time (Figure D), albeit with intra annual fluctuations. Since 2010, all districts have reported at least 1 SIAs per 6-month period (median 4, IQR (3-5)), with 16% of districts reporting >5. The exception is North Waziristan, where no SIAs took place between July to December 2012 and July to December 2013.

Spatio-temporal distribution of serotype-1 population immunity

Overall, estimates of population immunity to serotype-1 poliomyelitis in <36 month old children in Pakistan were nationally relatively high (74% of district with immunity >70%) from 2010 to 2016 (Fig 1, Fig 2 and Figure E). Immunity was estimated to be lower in FATA and Balochistan than the rest of the country and began to decline, particularly in these areas, from 2010 onwards reaching its lowest level in 2014 and 2011 in FATA and Balochistan, respectively (in the second half of 2011, 47% of the districts in Balochistan had estimates of immunity <50%, compared to 11% of the remaining districts; in the second half of 2014, 43% of the districts in FATA had estimates of immunity <50%, compared to 8% of the remaining districts). In particular, North and South Waziristan had the most dramatic decline, with estimates <30% throughout 2014. In July to December 2015, there were modest changes in population immunity. The most pronounced improvement was in the districts of Khyber and North and South Waziristan in FATA, with increases in population immunity of 11%, 14% and 6%, respectively. In January to June 2016, these three districts had improvements in population immunity of 13%, 20% and 15%, respectively. The improvements in July to December 2016 for these three districts were 7%, 14% and 8%, respectively.

Spatio-temporal distribution of RI coverage

Cohort coverage of three doses of tOPV delivered through RI was heterogeneous across Pakistan (Figure B). In general, RI coverage has been highest in in Punjab and lowest in FATA and Balochistan. This pattern has been consistent over time, with estimates of 85% (77-89%) in Punjab and 28% (19-43%) in FATA and Balochistan since January to June 2010. Since 2015, there have been modest improvements in RI coverage nationally, with estimates of 89% (86-92%) in Punjab and 34% (18-50%) in FATA and Balochistan. In the second half of 2016 (Fig 2), RI estimates were 90% (87-93%) in Punjab and in FATA and Balochistan, the estimates were 34% (18-56%).

Spatio-temporal distribution of SIA coverage

Cohort coverage of SIAs in the first half of 2010 was nationally relatively high, with estimates >100% in 19% of districts (Figure C). In 2012, there was a nationwide decline in SIA coverage, which remained consistent through to the first half of 2015. In July to December 2015, there was an overall improvement in coverage with 60% of districts reporting increases >10% (median 11%, IQR (7-14%)). In July to December 2016 (Fig 2), 11% of districts reported increases >10% (median 2%, IQR (0.6-5%)). Between January to June 2010 and July to December 2016, the lowest estimates in coverage were in FATA (median 48%, IQR (32-60%)) and the Quetta Block (i.e. Quetta, Killa Abdullah, Pishin) of Balochistan (median 38%, IQR (31-46%)).

S2 Estimation of force of infection (λ)

S2.1 Supplementary Methods

Description of the methods to estimate overall force of infection (FOI) (λ)

The FOI parameters α_1 and α_2 were jointly estimated as coefficients in the multivariable mixed effects model and separately in the univariable mixed effects model. The first component of the overall FOI refers to new infections as a result of ongoing transmission within a district and the second includes the spatial component (S_{ij}), which may be one of six spatial models detailed below, as defined by A_{ij}, R_{ij} and G_{ij} (i.e. S_{ij}=A_{ij}, S_{ij}=R_{ij} and S_{ij}=G_{ij}).

Description of the methods to estimate the spatial component of the transmission intensity

To look at the effects of population movement on the risk of infection, we used six spatial models to describe the connectivity between districts. In these models, each of N district administrative regions (i) forms one unit ($N = 140$).

1) Adjacency model

Firstly, we used an adjacency model (A_{ij}) to predict mobility patterns. Districts directly adjacent to district i were indexed with a value of 1 and all other districts with a value of 0. The movement to each district j from each district i were normalized to give the the probability of movement out of each district i.

2) Radiation model

Secondly, we used a radiation model (R_{ij}), to predict mobility patterns using a diffusion principle. The radiation model predicts the commuting flux from district i to district j and is dependent on the population sizes n_i and n_j and the population within a circle of radius equal to the Euclidean distance between the two populations s_{ij}[8]. The number of commuters moving from districts i to j (R_{ij}) is defined by:

$$R_{ij} = R_i * \left(\frac{n_i n_j}{(n_i + s_{ij}) * (n_i + n_j + s_{ij})} \right) \tag{3}$$

where R_i is the number of commuters moving out of district i. Estimating R_i was not possible in our context; we therefore, assumed a value of 1. Therefore, R_{ij} represents the probability of movement between districts i and j. The movement to each district j from each district i were normalized.

3) Radiation model (based on population density)

Thirdly, the radiation model was adapted to include population density instead of population size for values of n_i and s_{ij}.

4) Radiation model (based on driving time)

Fourthly, the radiation model was adapted to include driving time (in hours) extracted by Google maps (using ggmap R package) instead of Euclidean distance (d_{ij}) between all district pairs.

5) Gravity model

Fifthly, we used a gravity model (G_{ij}), which allows for a flexible dependence between each district pair i and j, driven by their respective population sizes (n_i and n_j) and the Euclidean distance (d_{ij}) between them.

$$G_{ij} = \frac{n_i^{\mu} * n_j^{\nu}}{d_{ij}^{\gamma}} \tag{4}$$

The parameters γ, μ, and ν are optimized exponents. These unknown parameters were optimized by maximizing the log-likelihood returned by the univariable mixed-effects logistic regression model. For the optimization of the parameters, we used the stochastic simulated-annealing method [9] in the optim function of the stats R package [10].

6) Gravity model fit to mobile phone data

Lastly, we used a gravity model (G_{ij}) fit to daily average number of trips between each i and j district pair. The daily average number of trips was extracted from mobile phone records between June 1 and December 31, 2013, previously published [11]. In this model we estimated coefficients for population size of each origin i, destination j and the Euclidean distances d_{ij} between them. The number of trips was assumed to be Poisson distributed with mean λ_{ij}, defined by the following model:

$$\begin{aligned} G_{ij} &\sim \text{Poisson}(\lambda_{ij}) \\ log(\lambda_{ij}) &= \alpha + \beta_1 \log(n_i) + \beta_2 \log(n_j) + \beta_3 \log(d_{ij}) \end{aligned} \tag{5}$$

where β_1, β_2 and β_3 are estimated coefficients. The fitted values were normalised by the sum of the origin i to all districts j, excluding within district movement.

S2.2 Supplementary Results

Results of force of infection (λ_j)

The six different spatial models of district connectivity provided varying fit to the district reported incidence of WPV serotype-1 cases, as evaluated by the log-likelihood, when incorporated into the univariable mixed-effects logistic regression model (Table D). The best fitting model incorporated the radiation model with population size into the force of infection from other districts (Fig 3 and Figure G). The probability of movement out of each origin district i is presented in (Figure I).

S3 Estimation of potential covariates

S3.1 Supplementary Methods

Estimation of non-polio AFP rate

Non-polio AFP rate per 100,000 population aged <15 years for each district and 6-month time period was estimated using the number of non-polio AFP cases and the district specific <15 year old population estimates. The district specific <15 year old population estimates were calculated based on the total population in a particular district (defined below) multiplied by the proportion <15 years of age based on the most recently available Pakistan Census [12].

Estimation of demographic variables (population size, population density, poverty, and total births)

Information on geographic variation in population size (2010), proportion of individuals living in poverty (as defined by the Multidimensional Poverty Index (MPI) from the Oxford Poverty & Human Development Initiative) (2007) [13] and total number of births (2010) was extracted from high-resolution maps from the Worldpop project [14]. These quantities are estimated at a resolution of 8.3e-3 decimal degrees. District-level estimates were calculated by aggregating the total population size and number of births, and taking the mean proportion of individuals living in poverty within district boundaries. The population density in each district was calculated as the population size per km^2.

Estimation of climatic variables (temperature and precipitation)

The mean annual temperature and precipitation for each district was estimated using data from the Climate Data Processing Centre (CDPC), within the Pakistan Meteorological Department (PMD) [15]. The data was based on mean annual rainfall in millimetres and mean daily temperatures in degrees Celsius between 1971 and 2000. More recent data was not publicly available.

S3.2 Supplementary Results

Spatio-temporal distribution of non-polio AFP rate

The non-polio AFP reporting rate per 100,000 population aged <15 years per six-month period has remained spatially heterogeneous since 2010, with 31% of districts reporting <1 AFP case per 100,000 per 6-month time period (median 1.46, IQR (0.82-2.36)) (Figure F). The province of Balochistan has consistently reported a rate of <1 per 100,000 (41% of district 6-month time periods). In North Waziristan between Jul-Dec 2013 to Jan-Jun 2014, the non-polio AFP rate increased to 18.26 from the previously reported 2.00 (2010 to first half of 2013). There are no distinct temporal changes in the overall spatial pattern of non-polio AFP.

Spatial distribution of demographic variables

Population size and density is greatest in Punjab and Sindh (median 1,593,000, IQR (1,354,000-2,647,000) and median 430.6 per km2, IQR (226.2-696.0)) and lowest in Balochistan (median 205,900, IQR (120,100-356,400) and median 22.6 per km^2, IQR (15.5-34.2)). The total number of births follows a similar pattern with the greatest number in Punjab and Sindh and fewest number in FATA and Balochistan. Adjusting the number of births for population size (i.e. birth rate per 100,000 population), results in a homogeneous spatial pattern (median 2584, IQR (2489-2678)). The proportion of people living in poverty is relatively high nationally in Pakistan (median 69%, IQR (55-78%)). The lowest proportion of poverty is in Punjab (median 54%, IQR (45-68%)) compared to the rest of

the country (median 71%, IQR (63-79%)). The spatial distribution of all demographic variables is presented in Figure H.

Spatial distribution of climatic variables

The temperature in Pakistan follows both a north-south and east-west gradient, with highest temperatures in the south-east and lowest temperatures in the north and west. The mean daily temperature range was 11-27 degrees Celsius. In Punjab and Sindh temperatures were high (median 25, IQR (25-25)); in the rest of the country, temperatures were lower (median 21, IQR (19-25)). Mean annual precipitation in Pakistan followed a north-south gradient with increased precipitation in northern districts. In Balochistan and Sindh the mean annual precipitation was much lower (median 100 mm, IQR (100-300)) than in FATA, KP, Islamabad, Gilgit-Baltistan and AJK (median 1,100 mm, IQR (500-1,300)). The spatial distribution of all climatic variables is presented in Figure H.

S4 Logistic regression model

S4.1 Supplementary Methods

We fit a series of univariable and multivariable generalized linear mixed-effects logistic regression models to the presence of poliomyelitis cases associated with wild poliovirus serotype-1 in districts over 6-month intervals from Jan-Jun 2010 to Jul-Dec 2016. The transmission intensity and a number of district-level characteristics were included as covariates, such as population immunity, routine immunization coverage, SIA coverage, population size and density, birth rate, poverty, and average annual temperature and precipitation. The model formulation for the probability of infection ($p_{i,t}$) in a district i at 6-month period t with a force of infection (λ_i) and q predictors ($X_{i1}...X_{iq}$) is:

$$\mu_{i,k} \sim \text{Normal}(0, \sigma_\mu^2)$$

$$\delta_k \sim \text{Normal}(0, \sigma_\delta^2)$$

$$\nu_t \sim \text{Normal}(0, \sigma_\nu^2)$$

$$\text{logit}(p_{i,k,t}) \sim \alpha + \beta_1 I_{i,t-1} + \beta_2 \sum_{i,i\neq j} I_{j,t-1} S_{ji} + ... + \beta_q X_{iq} + \beta_q X_{iqt} + \epsilon_i + \mu_{i,k} + \delta_k + \nu_t \qquad (6)$$

where α is the intercept, $\mu_{i,k}$ and δ_k are spatial-structured random effects at two different spatial levels (the district, μ, indexed by i, and the province, δ, indexed by k), and ν is a temporal random effect that accounts for a general time trend. The data was linked to the model in the following way:

$$X_{i,j,k} \sim \text{Binomial}(1, p_{i,k,t})$$

The model was fitted through maximum likelihood estimation and performed in the R programming language using the package lme4 [16].

S4.2 Supplementary Results

The spatial-structured random effects at the province level (δ) and the temporal random effect (6-month time period ν) are presented in Figure N.

S5 Supplementary Figures and Tables

Figure A: Map of Pakistan, with the first-level administrative boundaries and names of provinces. The publication of this map does not imply the expression of any opinion whatsoever on the part of WHO concerning the legal status of any territory, city or area or of its authorities, or concerning the delimitation of its frontiers or boundaries.

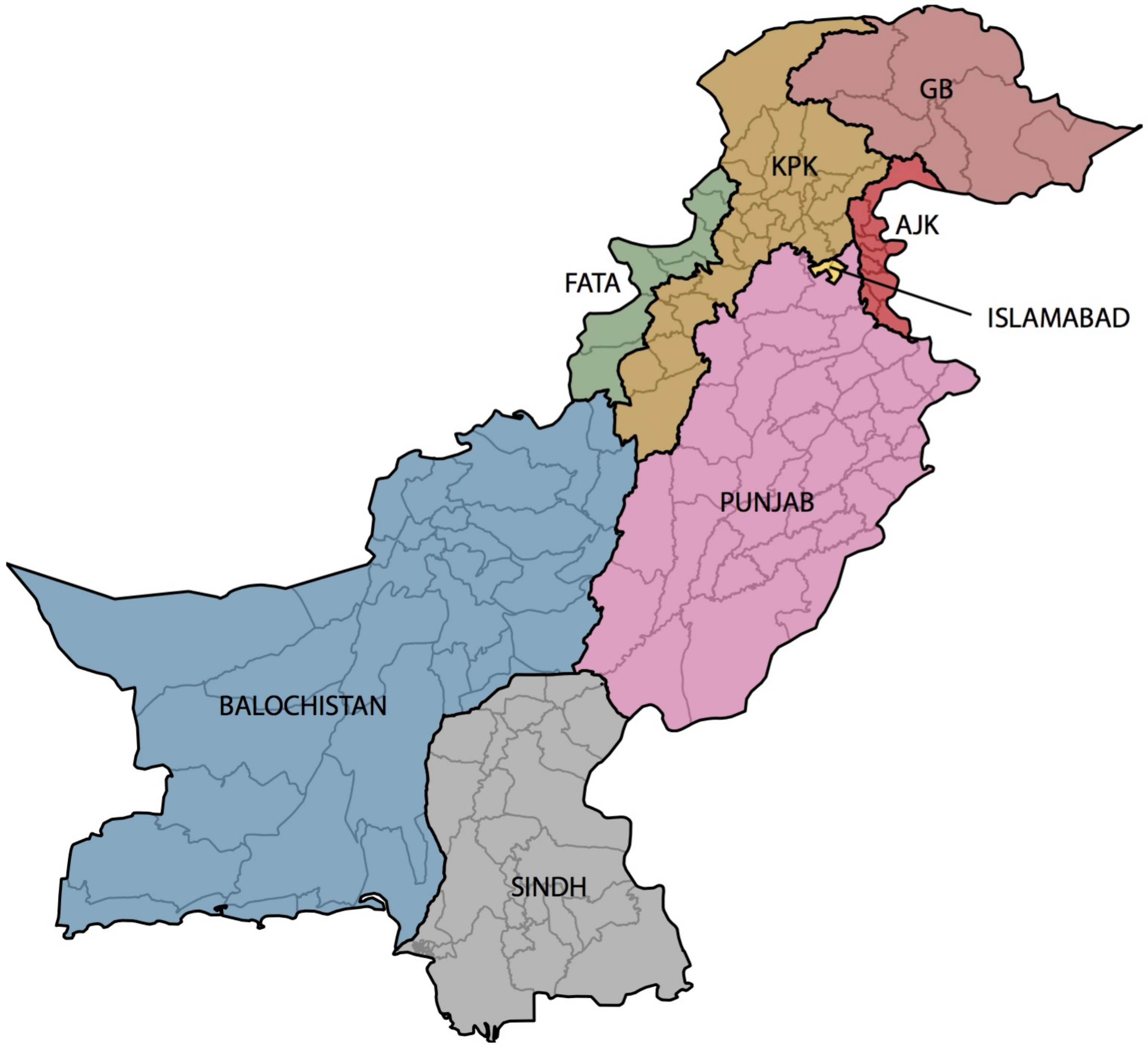

Figure B: Estimates of cohort routine immunisation coverage (%) for children <36 months in Pakistan (from 2010 to 2016). The publication of this map does not imply the expression of any opinion whatsoever on the part of WHO concerning the legal status of any territory, city or area or of its authorities, or concerning the delimitation of its frontiers or boundaries.

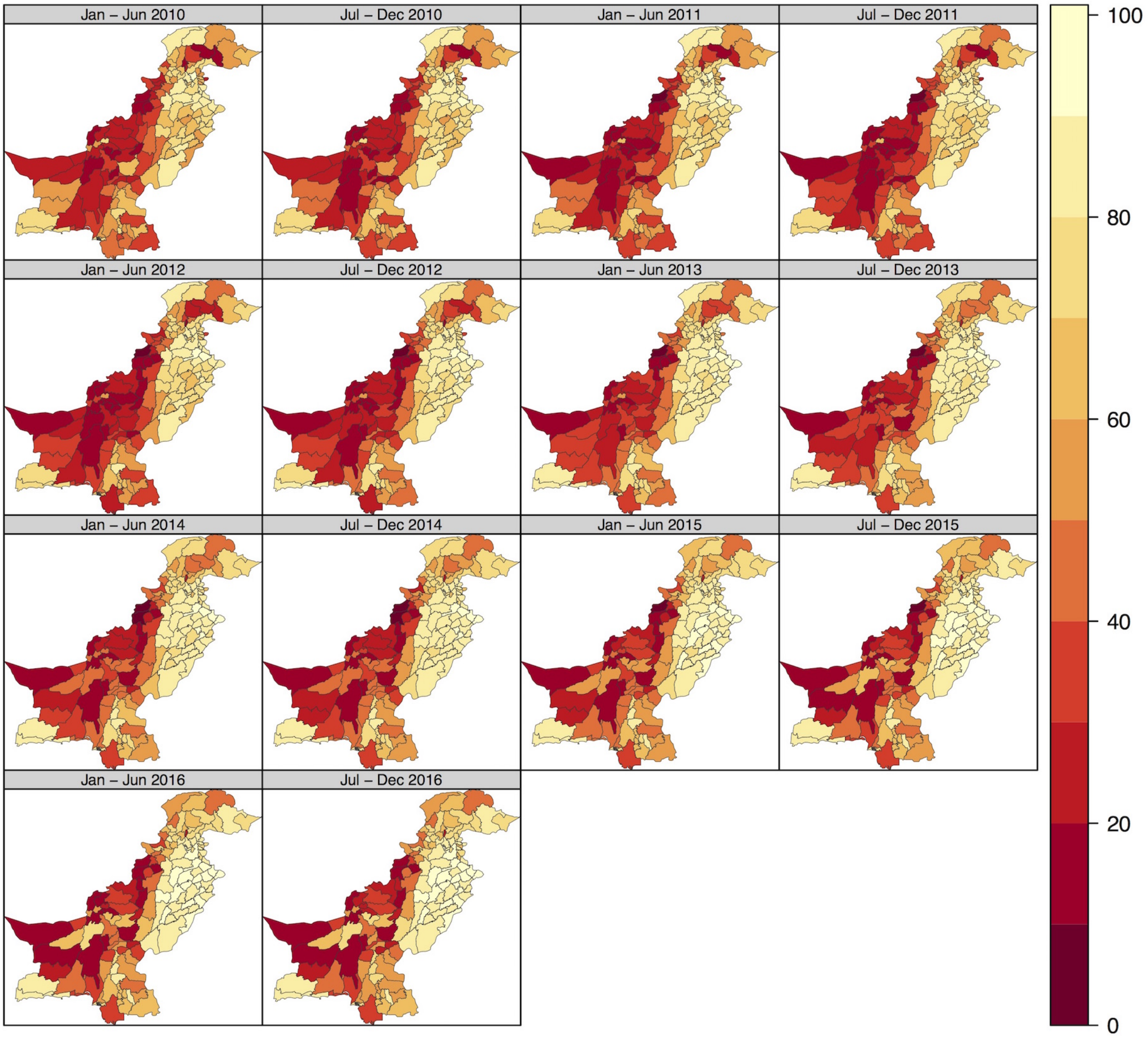

Figure C: Estimates of cohort SIA coverage (%) for children <36 months in Pakistan (from 2010 to 2016). The publication of this map does not imply the expression of any opinion whatsoever on the part of WHO concerning the legal status of any territory, city or area or of its authorities, or concerning the delimitation of its frontiers or boundaries.

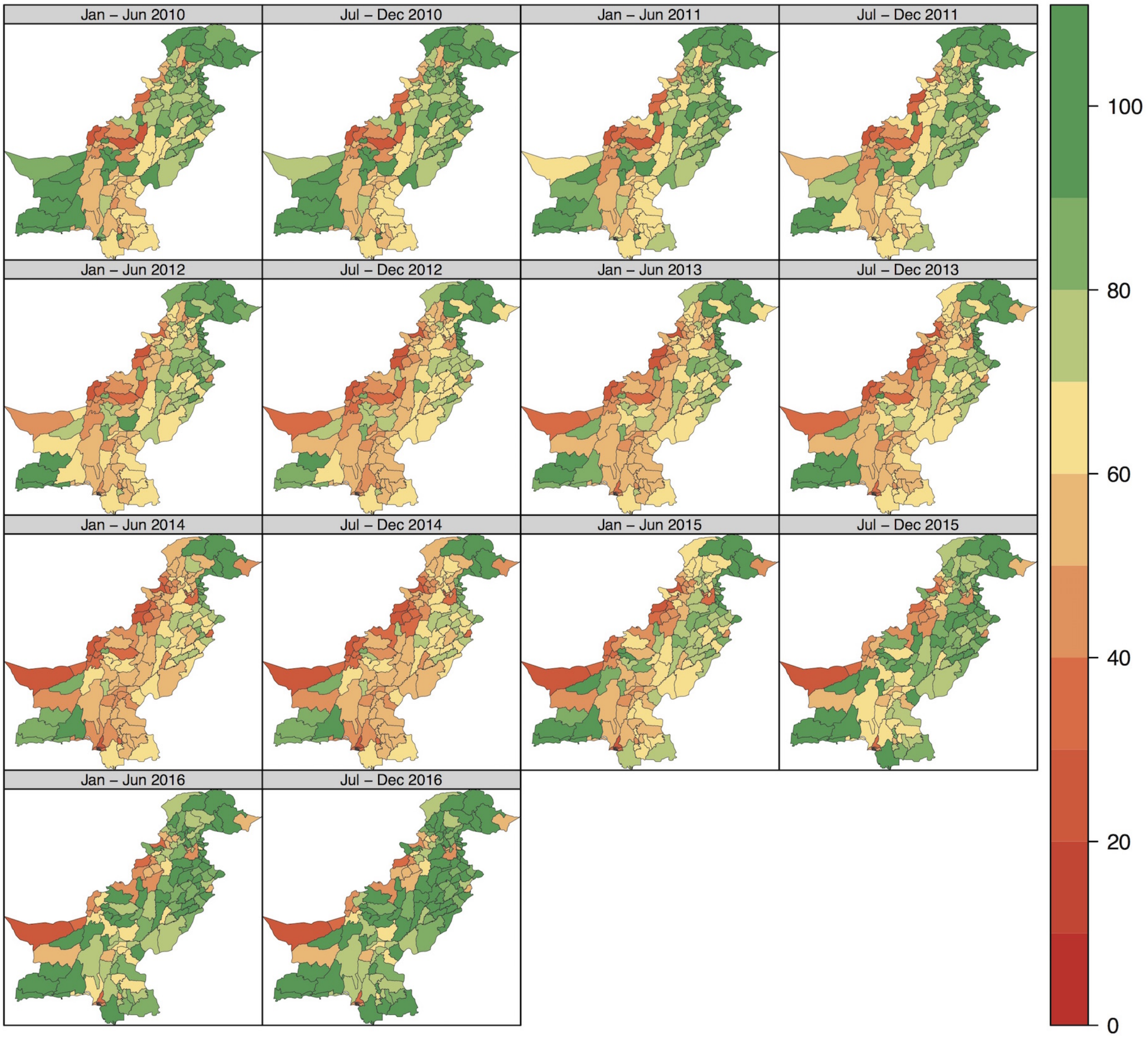

Figure D: Number of SIAs with serotype-1 oral poliovirus vaccine (OPV) (i.e. tOPV, bOPV and mOPV1) per district and 6-month period in Pakistan (from 2010 to 2016). Values greater than 6 are coloured at the same scale as 6 for ease of visualization. The publication of this map does not imply the expression of any opinion whatsoever on the part of WHO concerning the legal status of any territory, city or area or of its authorities, or concerning the delimitation of its frontiers or boundaries.

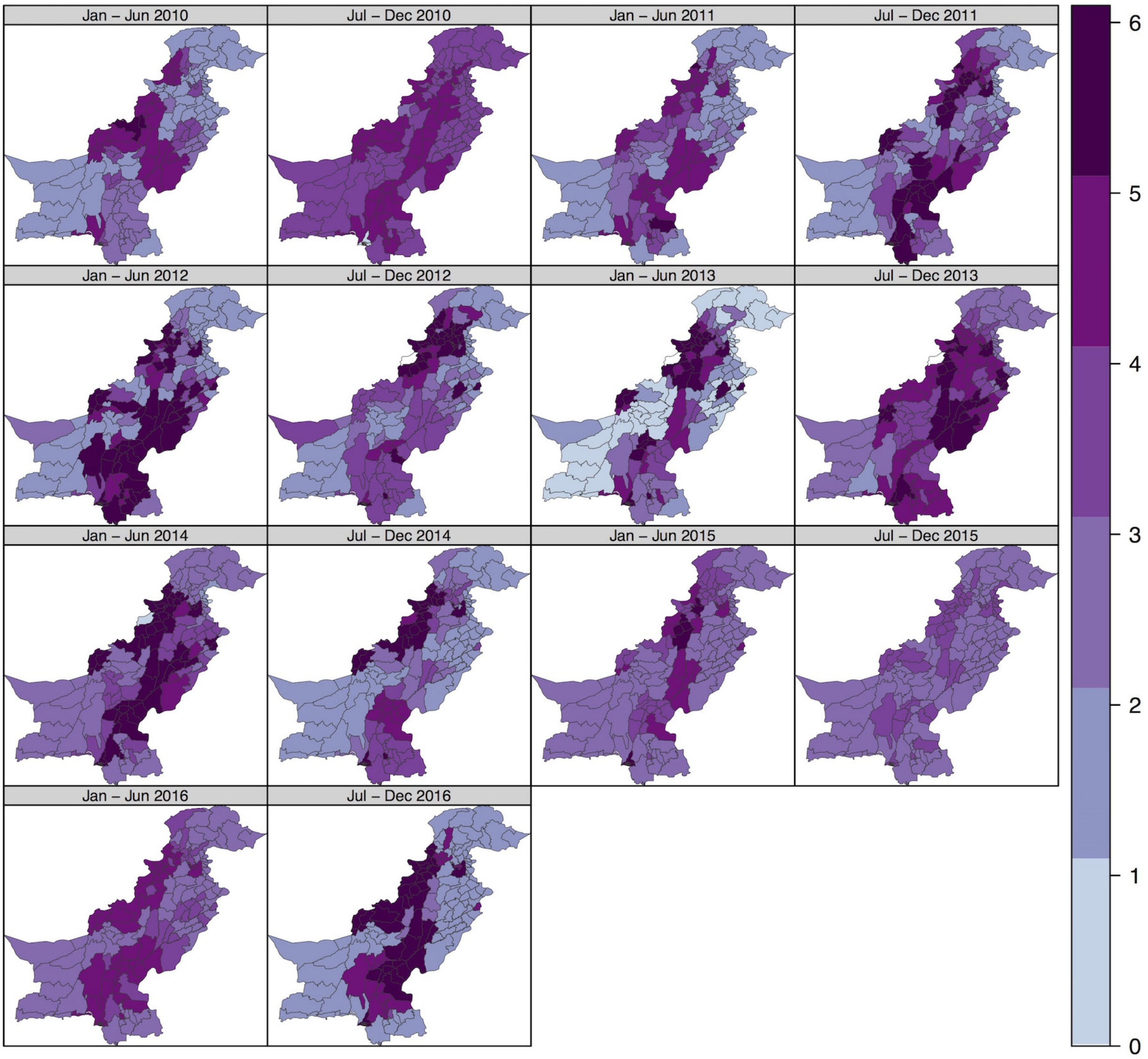

Figure E: Estimates of serotype-1 population immunity (%) against poliomyelitis for children <36 months in Pakistan (from 2010 to 2016). The publication of this map does not imply the expression of any opinion whatsoever on the part of WHO concerning the legal status of any territory, city or area or of its authorities, or concerning the delimitation of its frontiers or boundaries.

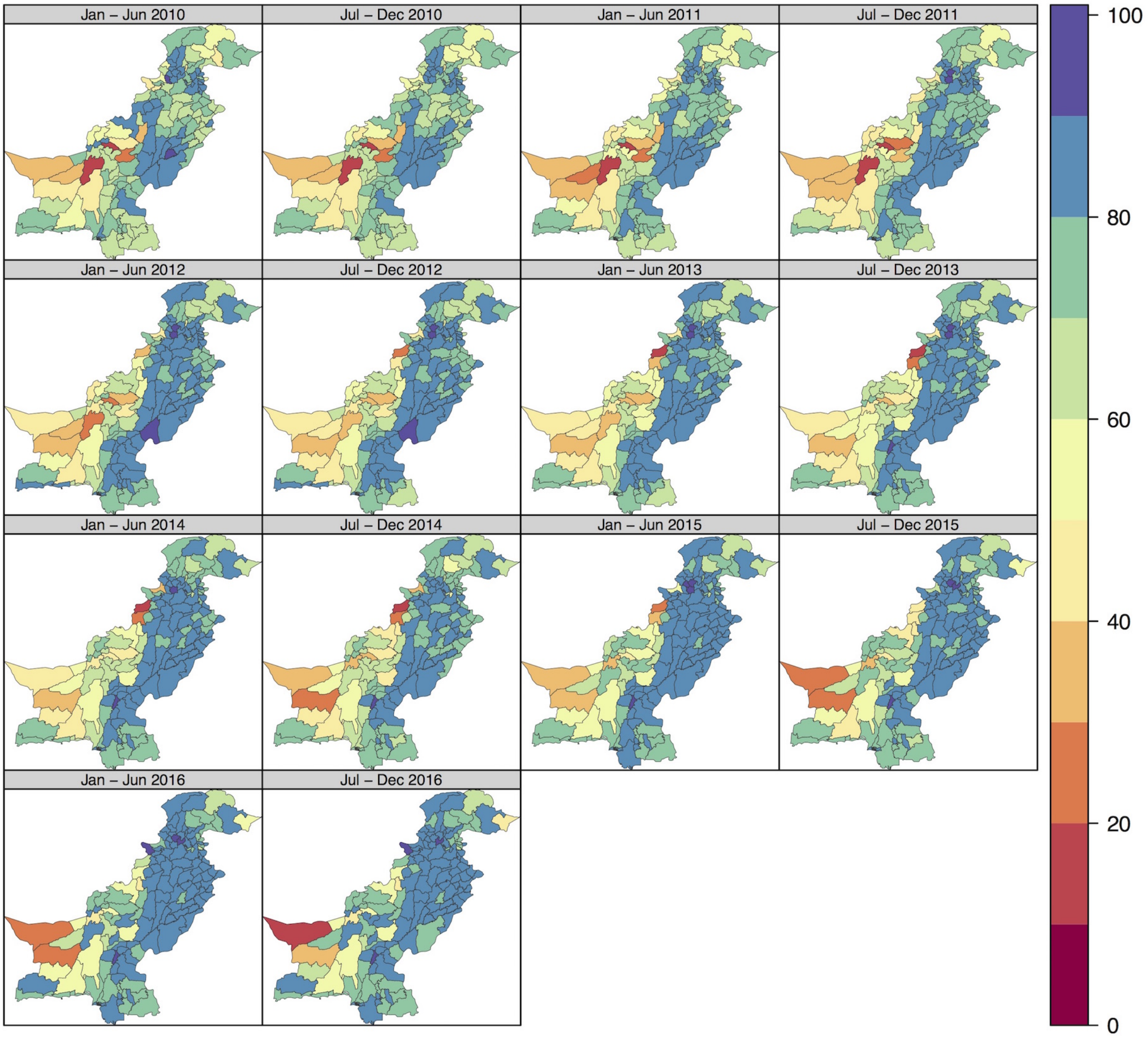

Figure F: Non-polio AFP reporting rate per 100,000 population <15 years in Pakistan (from 2010 to 2016). The publication of this map does not imply the expression of any opinion whatsoever on the part of WHO concerning the legal status of any territory, city or area or of its authorities, or concerning the delimitation of its frontiers or boundaries.

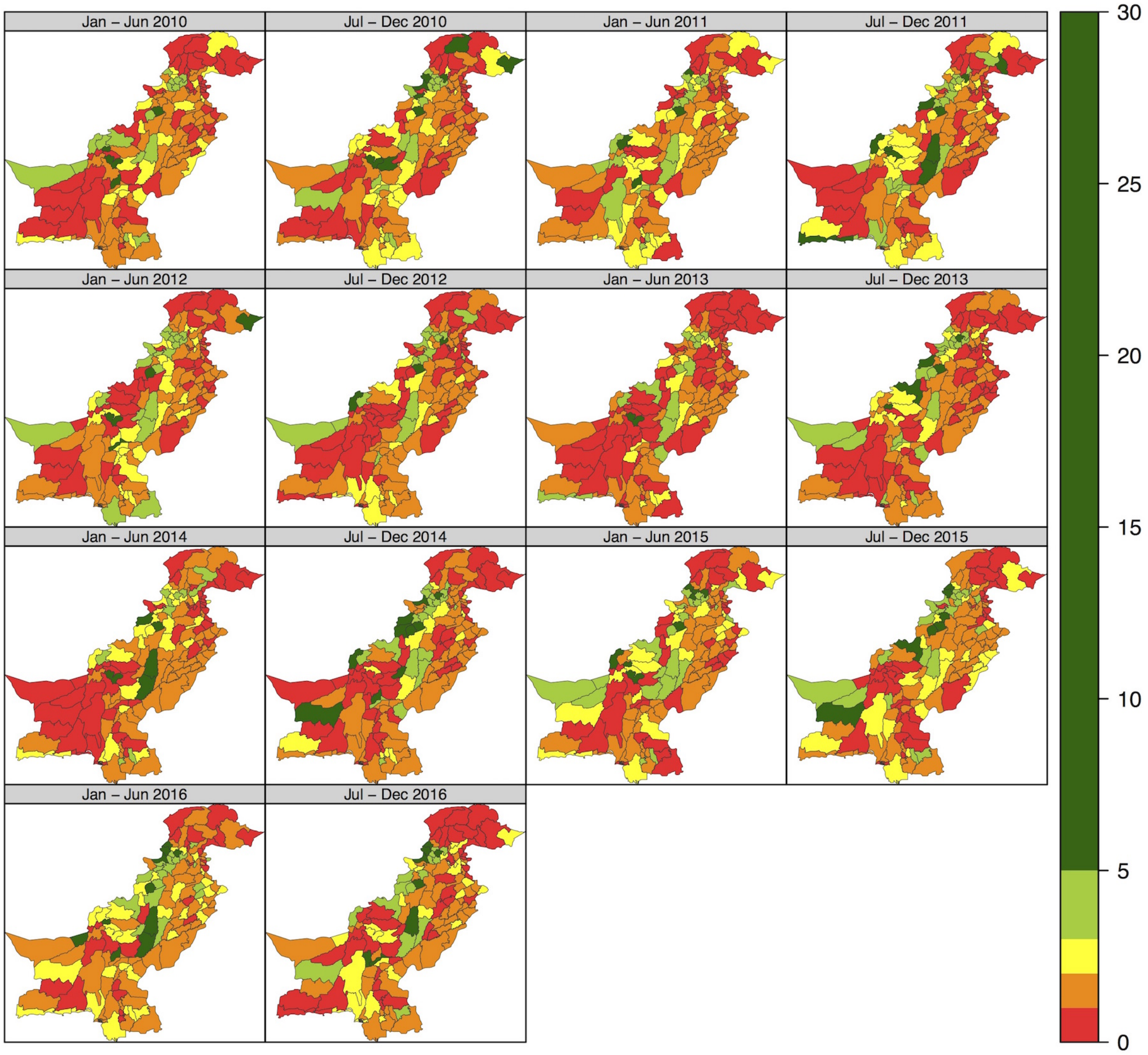

Figure G: Force of infection (incorporating the radiation model) in Pakistan (from July 2010 to June 2017). Values greater than 2 are coloured at the same scale as 2 for east of visualization. The publication of this map does not imply the expression of any opinion whatsoever on the part of WHO concerning the legal status of any territory, city or area or of its authorities, or concerning the delimitation of its frontiers or boundaries.

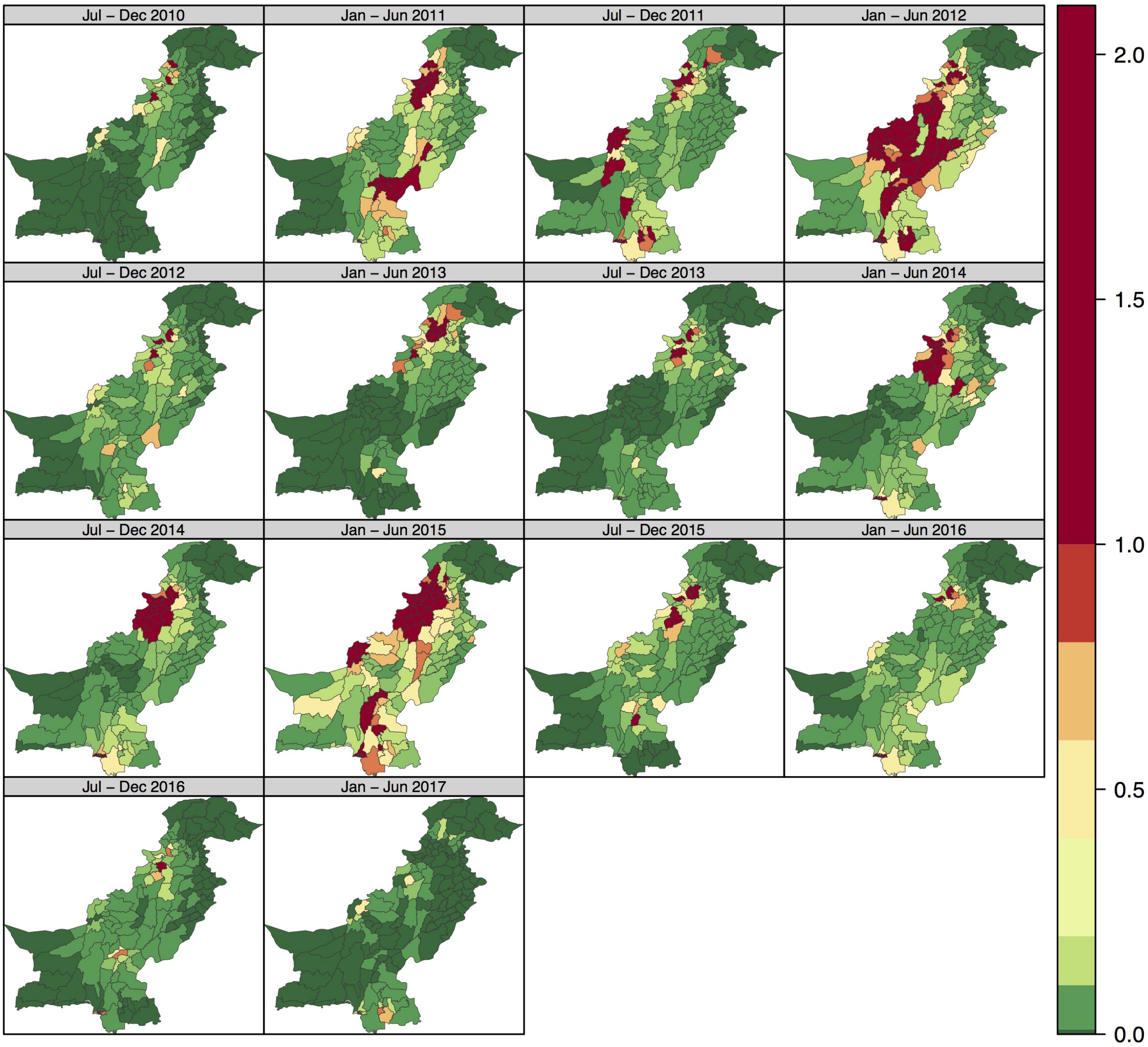

Figure H: Potential covariates of statistical model. The publication of this map does not imply the expression of any opinion whatsoever on the part of WHO concerning the legal status of any territory, city or area or of its authorities, or concerning the delimitation of its frontiers or boundaries.

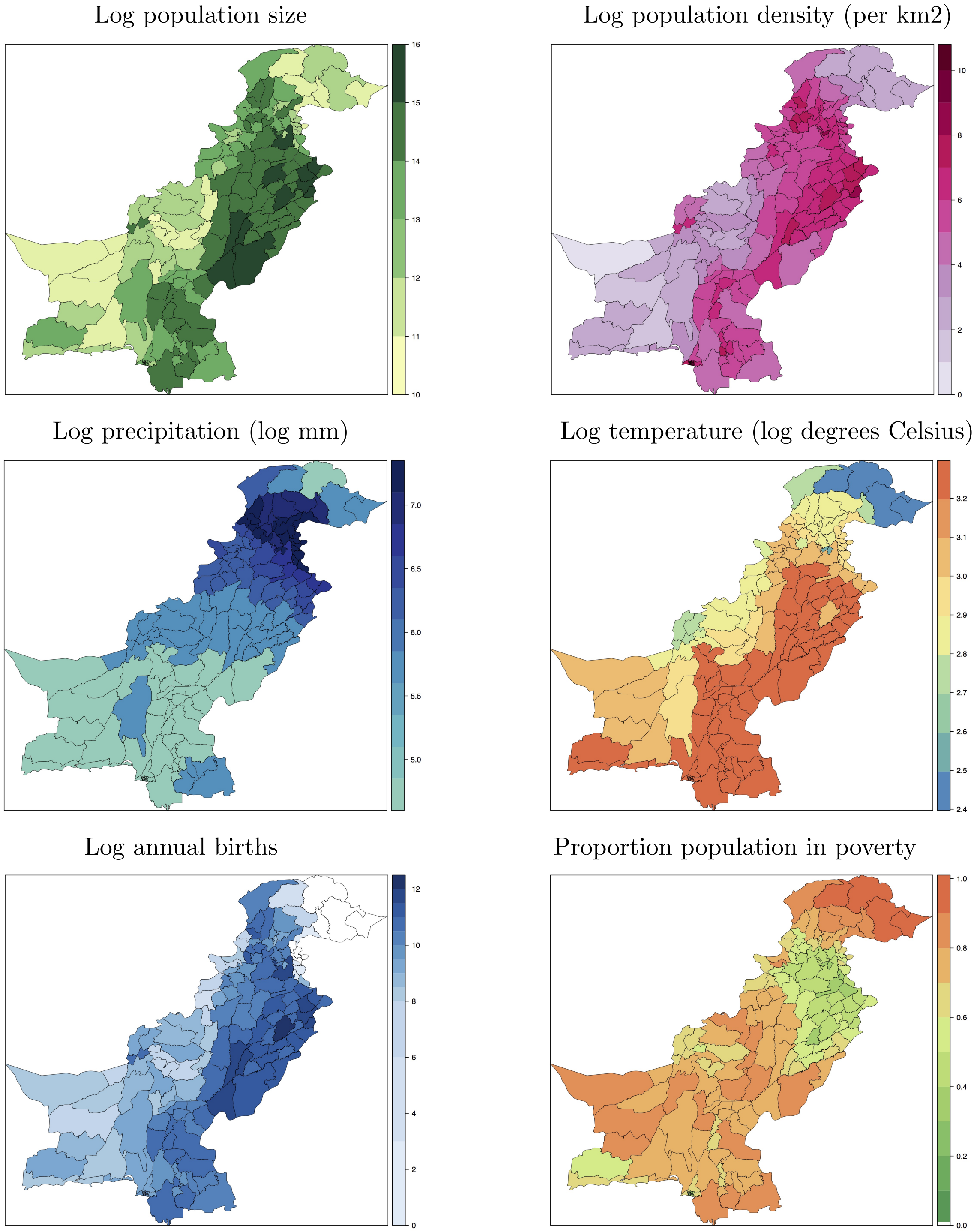

Figure I: Probability of movement out of each district in Pakistan based on the radiation model. The name per panel and dark blue shading indicates the origin district. The publication of this map does not imply the expression of any opinion whatsoever on the part of WHO concerning the legal status of any territory, city or area or of its authorities, or concerning the delimitation of its frontiers or boundaries.

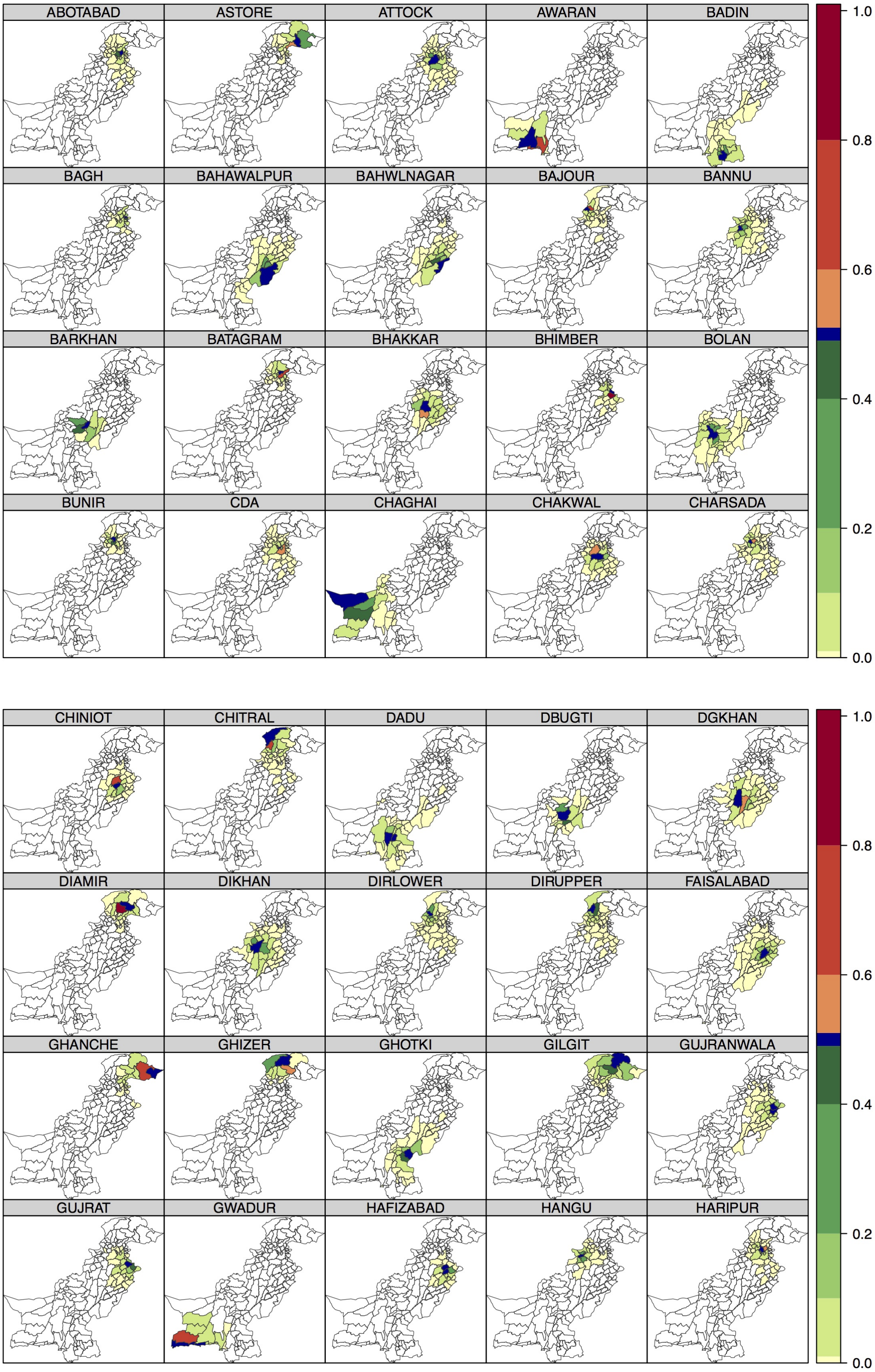

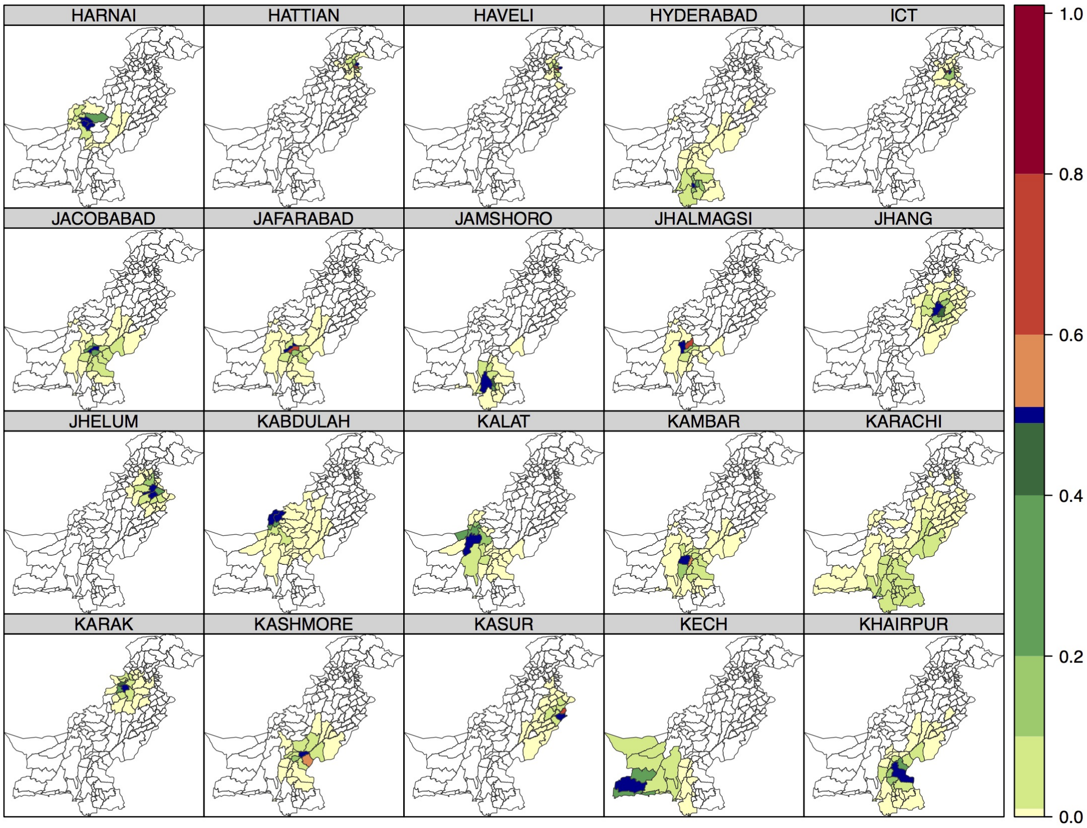
HARNAI
HATTIAN
HAVELI
HYDERABAD
ICT
JACOBABAD
JAFARABAD
JAMSHORO
JHALMAGSI
JHANG
JHELUM
KABDULAH
KALAT
KAMBAR
KARACHI
KARAK
KASHMORE
KASUR
KECH
KHAIRPUR
1.0
0.8
0.6
0.4
0.2
0.0

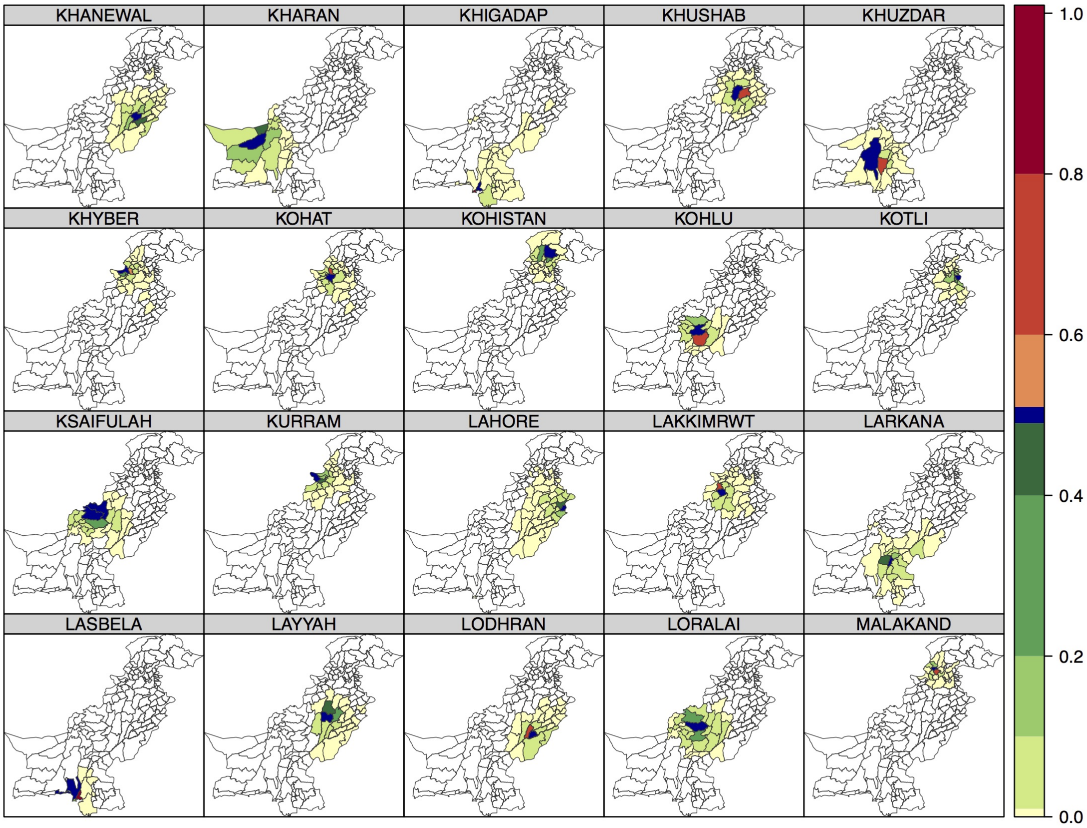
KHANEWAL
KHARAN
KHIGADAP
KHUSHAB
KHUZDAR
KHYBER
KOHAT
KOHISTAN
KOHLU
KOTLI
KSAIFULAH
KURRAM
LAHORE
LAKKIMRWT
LARKANA
LASBELA
LAYYAH
LODHRAN
LORALAI
MALAKAND
1.0
0.8
0.6
0.4
0.2
0.0

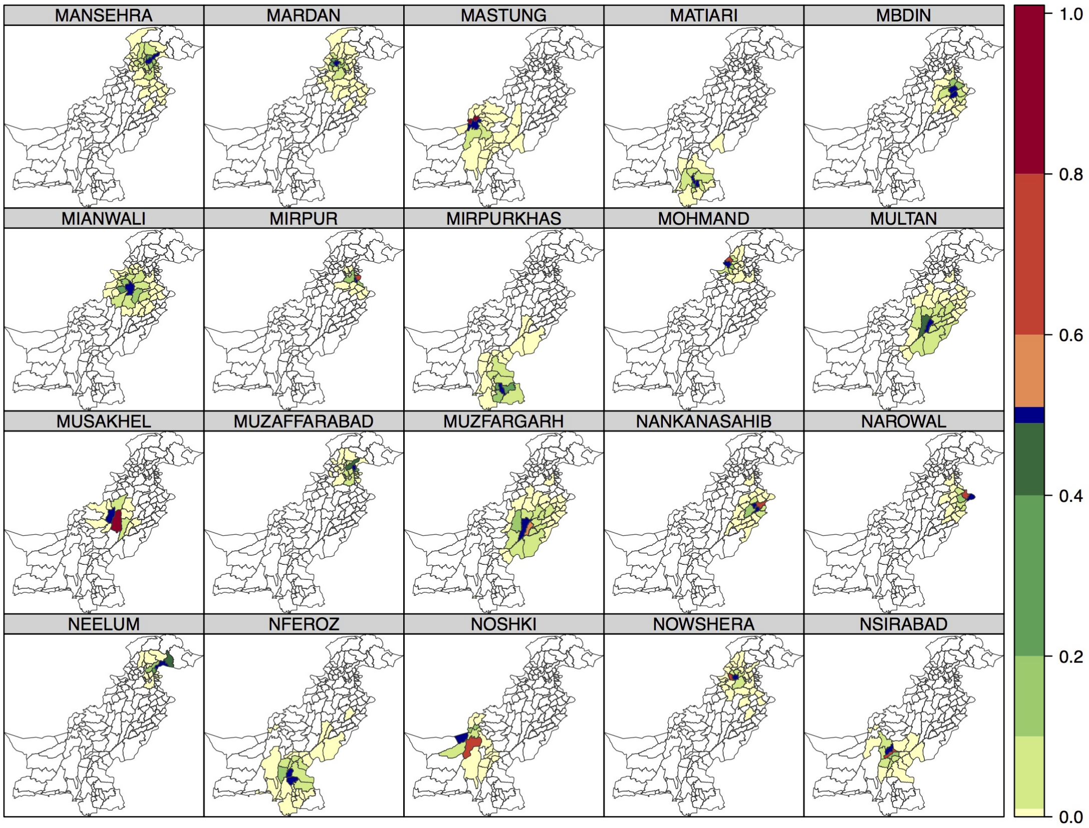
MANSEHRA
MARDAN
MASTUNG
MATIARI
MBDIN
MIANWALI
MIRPUR
MIRPURKHAS
MOHMAND
MULTAN
MUSAKHEL
MUZAFFARABAD
MUZFARGARH
NANKANASAHIB
NAROWAL
NEELUM
NFEROZ
NOSHKI
NOWSHERA
NSIRABAD
1.0
0.8
0.6
0.4
0.2
0.0

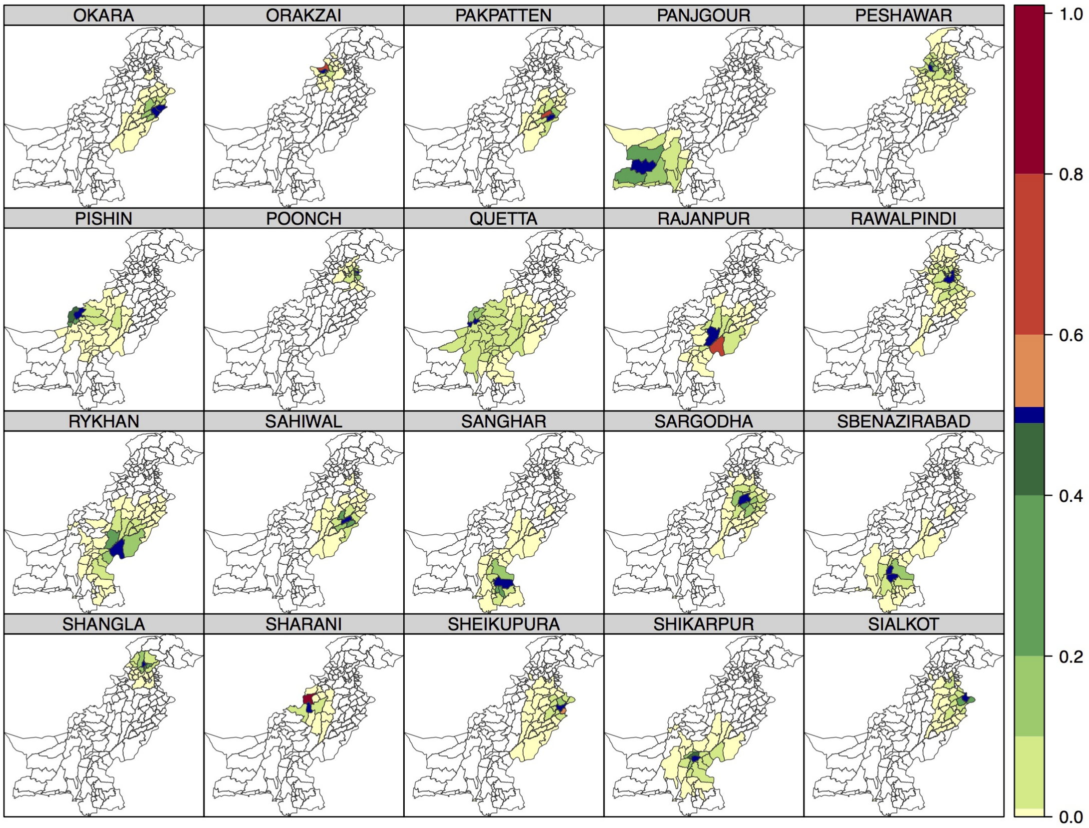
OKARA
ORAKZAI
PAKPATTEN
PANJGOUR
PESHAWAR
PISHIN
POONCH
QUETTA
RAJANPUR
RAWALPINDI
RYKHAN
SAHIWAL
SANGHAR
SARGODHA
SBENAZIRABAD
SHANGLA
SHARANI
SHEIKUPURA
SHIKARPUR
SIALKOT
1.0
0.8
0.6
0.4
0.2
0.0

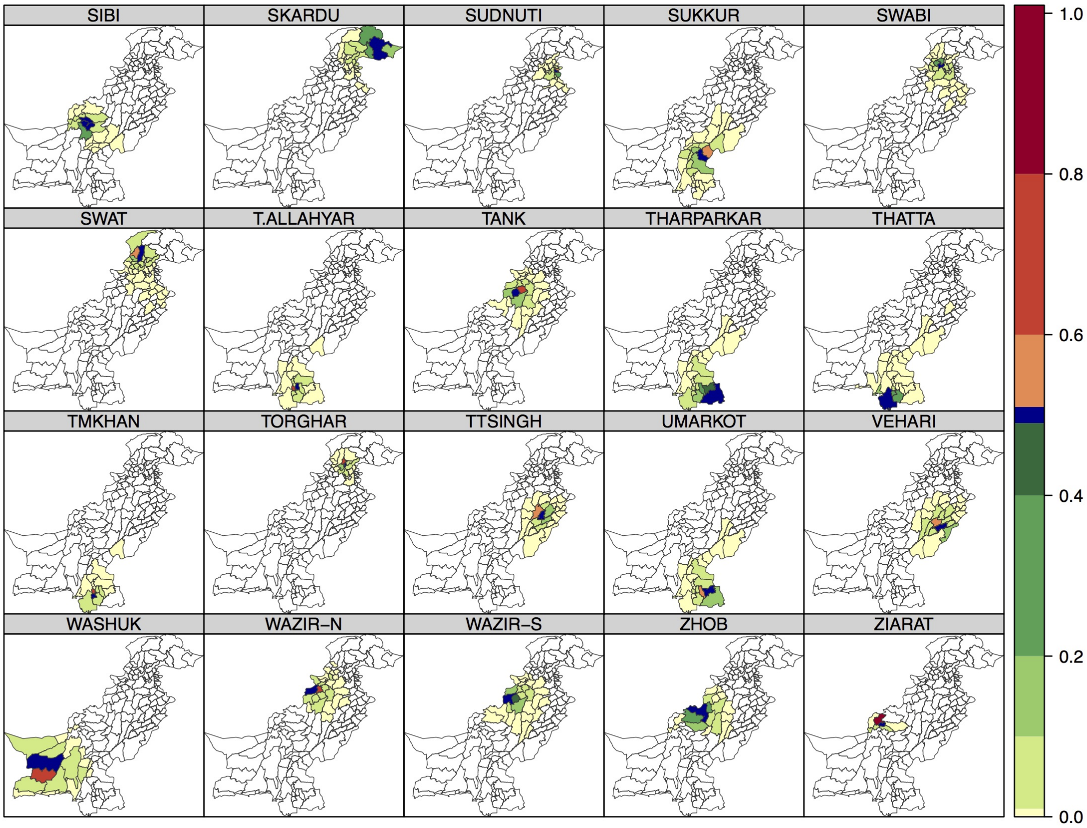
SIBI
SKARDU
SUDNUTI
SUKKUR
SWABI
SWAT
T.ALLAHYAR
TANK
THARPARKAR
THATTA
TMKHAN
TORGHAR
TTSINGH
UMARKOT
VEHARI
WASHUK
WAZIR−N
WAZIR−S
ZHOB
ZIARAT
1.0
0.8
0.6
0.4
0.2
0.0

Figure J: Cumulative probability of movement out of each district (based on the radiation model) by Euclidian distance (km). The cumulative probability moving out of Karachi and North Waziristan are highlighted in red and green, respectively.

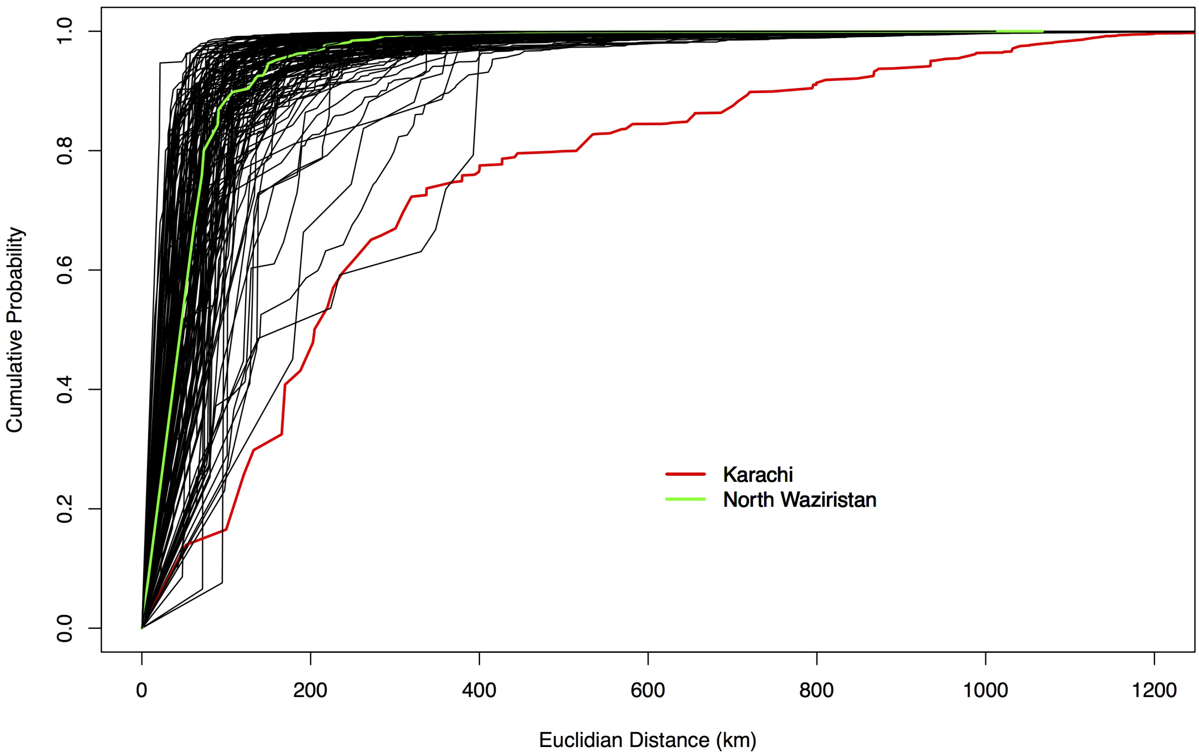

Figure K: Observed wild poliovirus serotype-1 cases in Pakistan (from 2010 to 2016). The publication of this map does not imply the expression of any opinion whatsoever on the part of WHO concerning the legal status of any territory, city or area or of its authorities, or concerning the delimitation of its frontiers or boundaries.

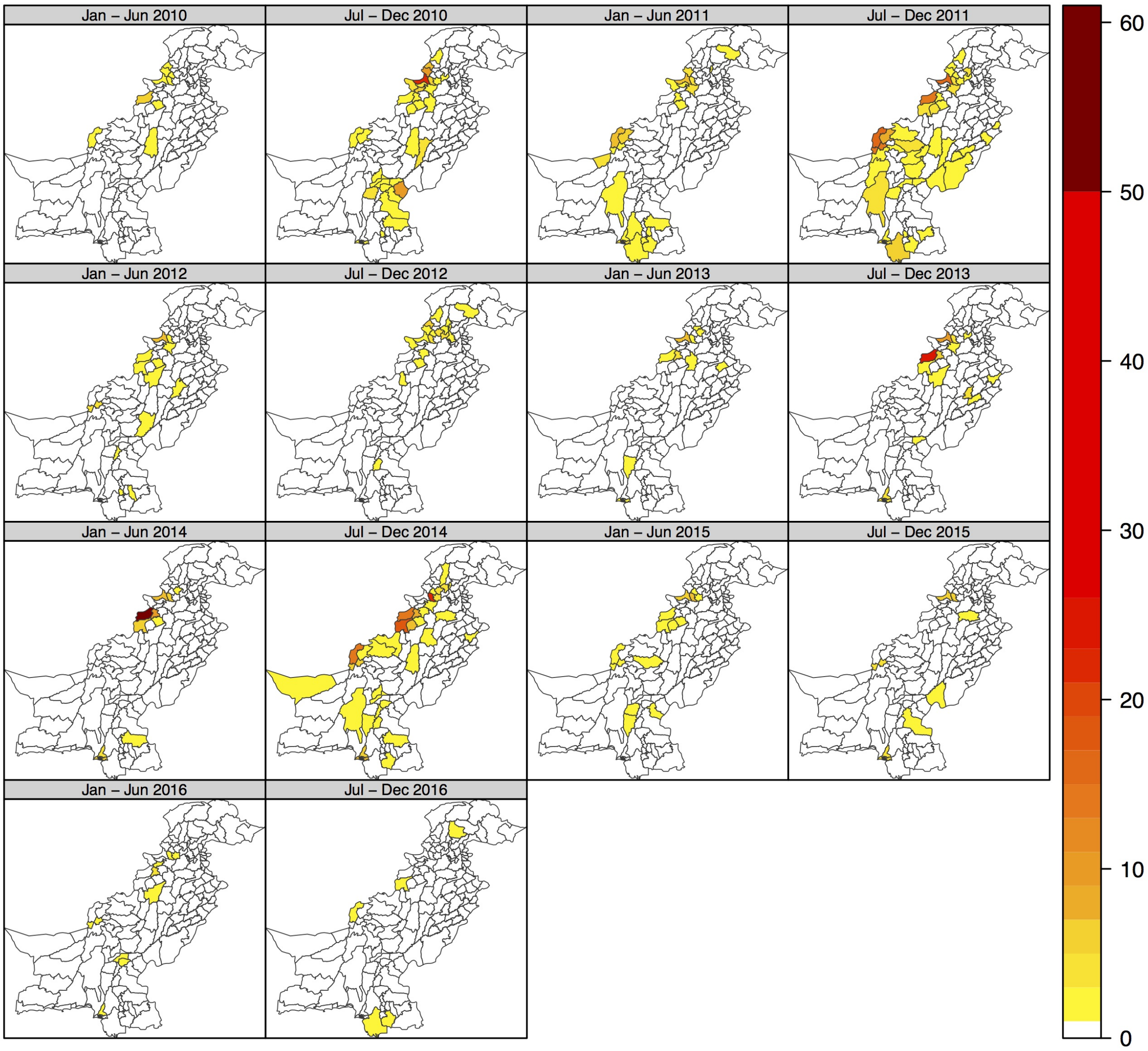

Figure L: Probability of reporting wild poliovirus serotype-1 cases in Pakistan (from 2010 to 2016) based on the best fitting logistic regression model. The publication of this map does not imply the expression of any opinion whatsoever on the part of WHO concerning the legal status of any territory, city or area or of its authorities, or concerning the delimitation of its frontiers or boundaries.

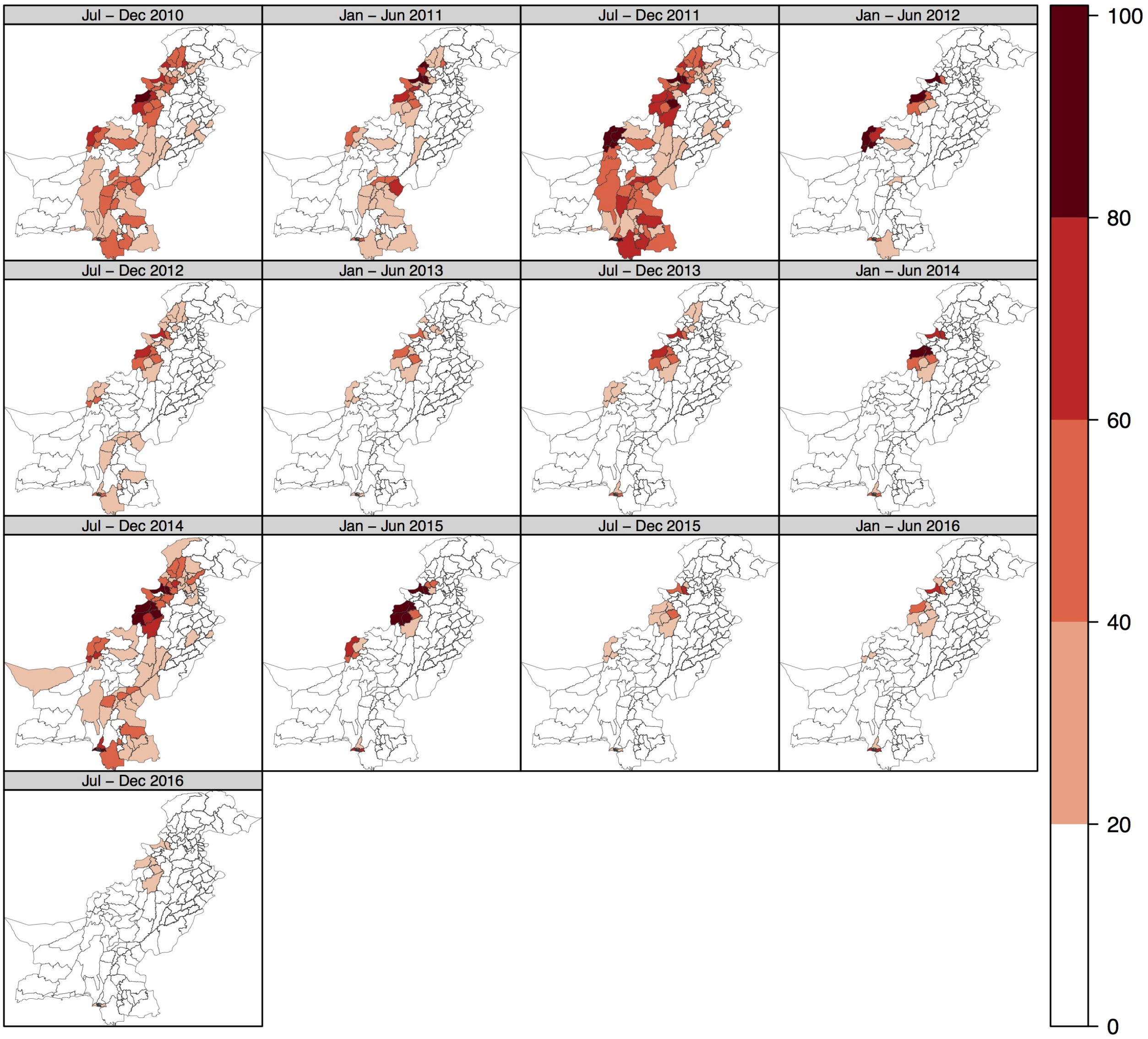

Figure M: Performance of model prediction. ROC for each 6-month prediction of districts with serotype-1 poliovirus cases between July - Dec 2013 and July - Dec 2016. The legends highlights varying probability thresholds.

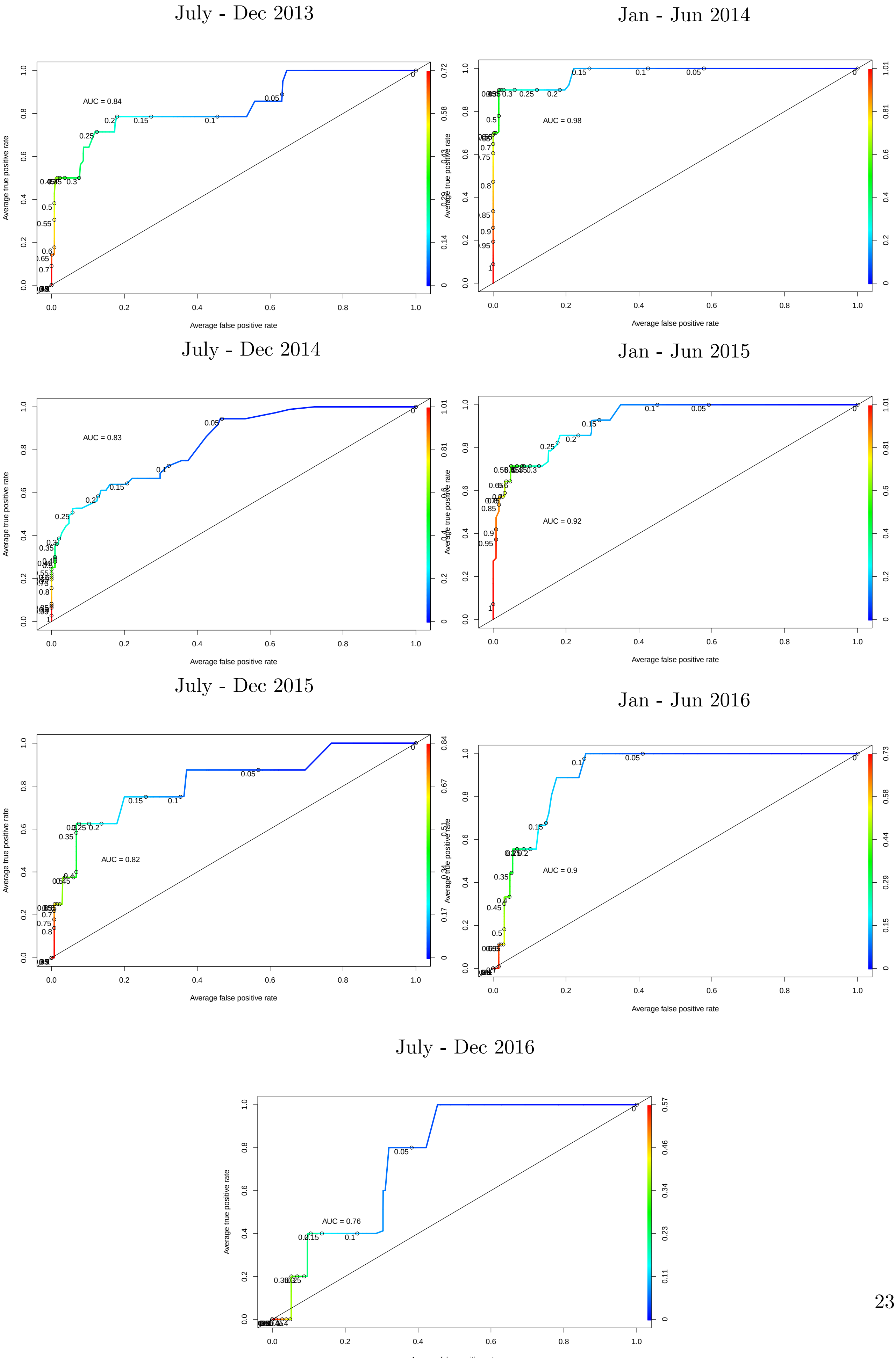

Figure N: Random effect estimates for province and time (6-month period) from best fitting model. The publication of this map does not imply the expression of any opinion whatsoever on the part of WHO concerning the legal status of any territory, city or area or of its authorities, or concerning the delimitation of its frontiers or boundaries.

Random effect of province

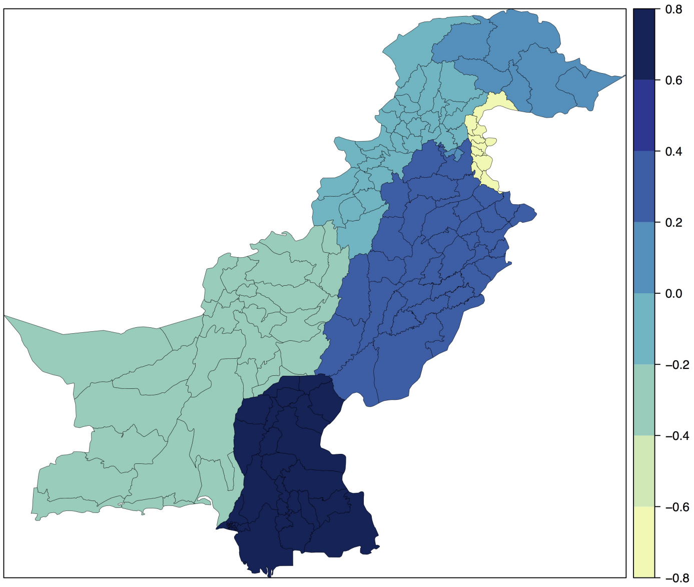

Random effect of time (6-month period)

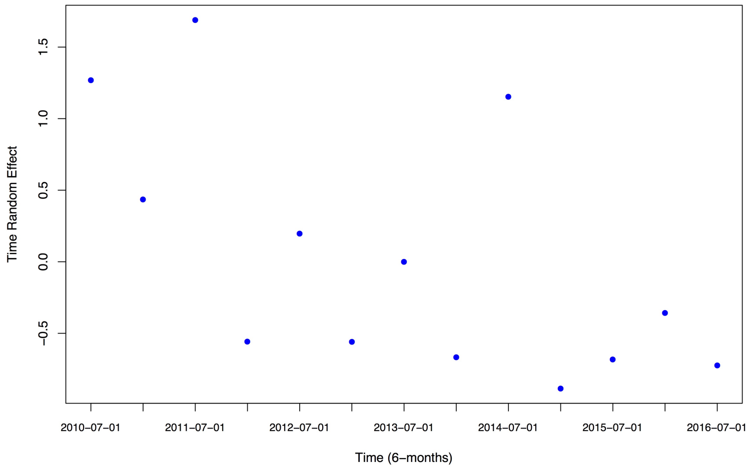

Table A: Parameter estimates (mean and 95% credible interval) for the spatio-temporal random-effects models of serotype-1 population immunity, Routine immunization coverage and SIA coverage. For SIA coverage, the gamma distribution is assumed, as indicated by the asterisk (*).

Parameters	Serotype-1 population immunity Mean (95% CI)	Routine immunization coverage Mean (95% CI)	SIA coverage Mean (95% CI)
Intercept, α	0.40 (-0.07–0.87)	0.14 (-0.32–0.61)	-0.14 (-0.60–0.32)
Precision Gaussian observations, $1/\sigma^2$	21.13 (21.06–21.18)	3.86 (3.81–3.92)	0.76 (0.71–0.81)*
Precision district-time interaction, $1/\sigma_\gamma^2$	103.62 (103.28–103.95)	88.97 (86.77–90.55)	2825.21 (1021.21–6791.59)
Precision province-time interaction, $1/\sigma_\delta^2$	120.48 (119.92–120.93)	90.64 (86.67–93.22)	9888.92 (481.86–40490.55)
Precision general temporal trend, $1/\sigma_\omega^2$	66.72 (66.50–66.90)	68.90 (67.73–69.88)	194.77 (69.28–428.82)
Precision spatially-unstructured effect, $1/\sigma_\mu^2$	147.39 (146.90–147.87)	68.67 (67.64–70.00)	1843.02 (132.31–6689.94)
Precision spatially-structured effect, $1/\sigma_\nu^2$	66.04 (66.50–66.90)	52.54 (51.70–53.32)	1901.19 (134.89–6765.24)

Table B: Univariable analysis (Odds Ratio and 95% confidence interval) for the relationship between observed wild poliovirus serotype-1 cases and potential covariates in 6-month time intervals between Jan - June 2010 and July - Dec 2016. OR and 95% CI for population immunity, routine immunization and SIA coverage is for a 10% increase. Random effects of province, district and time were included in the univariable analysis. SIA=Supplementary Immunization Activity; AFP=acute flaccid paralysis; and FOI=force of infection.

Variables	Odds Ratio (95% CI)	P-value	logLik
Serotype-1 Population Immunity (previous 6-months)	0.71 (0.59–0.85)	<0.001	-547.8
Routine Immunization coverage (previous 6-months)	0.73 (0.63–0.83)	<0.001	-544.1
SIA coverage (previous 6-months)	0.59 (0.51–0.69)	<0.001	-528.4
SIA number1 (2-4 versus 0-1)	0.19 (0.06–0.58)	0.003	-549.6
SIA number1 ($\geq$ 5 versus 0-1)	0.32 (0.10–0.96)	0.043	
SIA number2 (2-4 versus 0-1)	0.19 (0.06–0.57)	0.003	-549.3
SIA number2 (5-6 versus 0-1)	0.29 (0.09–0.92)	0.035	
SIA number2 ($\geq$ 7 versus 0-1)	0.36 (0.11–1.14)	0.082	
Non-polio AFP rate (previous 6-months)	1.19 (1.07–1.32)	0.001	-549.5
Population size (log)	2.28 (1.49–3.50)	<0.001	-569.4
Population density (population per km^2)	1.46 (1.11–1.93)	0.007	-573.0
Log(Temperature) (average daily, degrees Celsius)	1.39 (0.14–13.96)	0.781	-576.3
Log(Precipitation) (average annual, millimetres)	0.62 (0.36–1.05)	0.075	-574.8
Log(Births)	1.26 (1.02–1.55)	0.032	-574.2
Log(Birth rate) per 100,000 population	1.02 (0.73–1.41)	0.916	-576.4
Poverty (Multidimensional Poverty Index)	0.45 (0.05–4.14)	0.483	-576.1
FOI (current district)	1.30 (1.16–1.45)	<0.001	-540.3
FOI (between districts - Adjacency model)	1.10 (1.00–1.21)	0.049	-552.6
FOI (between districts - Radiation model)	1.22 (1.08–1.38)	0.001	-548.3
FOI (between districts - Radiation model (population density))	1.13 (1.03–1.24)	0.011	-550.7
FOI (between districts - Radiation model (travel times))	1.07 (0.99–1.17)	0.102	-553.2
FOI (between districts - Gravity model (mobile phone data))	0.97 (0.74–1.28)	0.836	-554.5
FOI (between districts - Gravity model (optimised parameters))	0.76 (0.57–1.01)	0.063	-552.4

Table C: Liklihood ratio (LR) test comparing multivariable model with force of infection (FOI) between districts based on radiation model versus no FOI between districts for 6-month ahead out of sample predictions.

Time period for prediction	Log likelihood (with FOI)	Log likelihood (without FOI)	LR test p-value
July-Dec 2013	-284.98	-285.88	0.179
Jan-Jun 2014	-319.40	-320.36	0.165
July-Dec 2014	-335.14	-337.09	0.048
Jan-Jun 2015	-393.51	-395.98	0.026
July-Dec 2015	-420.41	-423.01	0.022
Jan-Jun 2016	-446.13	-448.70	0.023
July-Dec 2016	-471.17	-473.85	0.021

Table D: Comparison of area under the curve (AUC) for 6-month ahead out of sample predictions using the multivariable model without force of infection (FOI) between districts, with FOI based on radiation model and with FOI based on adjacency model.

Time period for prediction	AUC (FOI rad)	AUC (FOI adj)	AUC (without FOI)
July-Dec 2013	0.84	0.86	0.84
Jan-Jun 2014	0.98	0.97	0.98
July-Dec 2014	0.83	0.82	0.83
Jan-Jun 2015	0.92	0.92	0.92
July-Dec 2015	0.82	0.82	0.81
Jan-Jun 2016	0.90	0.90	0.91
July-Dec 2016	0.76	0.76	0.76

References

[1] O'Reilly, K. M. *et al.* A new method to estimate the coverage of mass vaccination campaigns against poliomyelitis from surveillance data. *American Journal of Epidemiology* (2015).

[2] Grassly, N. C. *et al.* New strategies for the elimination of polio from India. *Science* **314**, 1150–1153 (2006).

[3] Mangal, T. D. *et al.* Key issues in the persistence of poliomyelitis in Nigeria: a case-control study. *The Lancet Global Health* **2**, e90–e97 (2014).

[4] Rue, H., Martino, S. & Chopin, N. Approximate Bayesian inference for latent Gaussian models by using integrated nested Laplace approximations. *Journal of the Royal Statistical Society: Series B* **71**, 319–392 (2009).

[5] R-INLA. URL www.r-inla.org.

[6] Pons-Salort, M. *et al.* Population immunity against serotype-2 poliomyelitis leading up to the global withdrawal of the oral poliovirus vaccine: Spatio-temporal modelling of surveillance data. *PLoS Medicine* **13** (2016).

[7] Besag, J., York, J. & Mollie, A. Bayesian Image-Restoration, with 2 Applications in Spatial Statistics. *Annals of the Institute of Statistical Mathematics* **43**, 1–20 (1991).

[8] Simini, F., Gonzalez, M. C., Maritan, A. & Barabasi, A.-L. A universal model for mobility and migration patterns. *Nature* **484**, 96–100 (2012).

[9] Bertsimas, D. & Tsitsiklis, J. Simulated Annealing. *Statistical Science* **8** (1993).

[10] R Core Team. *R: A Language and Environment for Statistical Computing*. R Foundation for Statistical Computing, Vienna, Austria (2015). URL https://www.R-project.org/.

[11] Wesolowski, A. *et al.* Impact of human mobility on the emergence of dengue epidemics in Pakistan. *Proc Natl Acad Sci* **112**, 11887–92 (2015).

[12] Pakistan Census. Population by selective age groups. 1988. URL www.pbs.gov.pk/content/population-selective-age-groups.

[13] Multidimensional Poverty Index. Oxford Poverty and Human Development Initiative. University of Oxford. URL www.ophi.org.uk/policy/multidimensional-poverty-index/.

[14] WorldPop Project. URL www.worldpop.org.uk.

[15] Climate Data Processing Centre. Pakistan Meteorological Department. Karachi, Pakistan. URL www.pmd.gov.pk/cdpc/home.htm.

[16] Bates, D., Mächler, M., Bolker, B. & Walker, S. Fitting linear mixed-effects models using lme4. *Journal of Statistical Software* **67**, 1–48 (2015).
